# Valorization of Rice Husk through the Development of New Materials of Silica Functionalized with Phthaloyl Diamide Ligands: Characterization, Isomer Effect, Ag^+^ Adsorption Investigation, and Antibacterial Activity

**DOI:** 10.1021/acsomega.6c04955

**Published:** 2026-10-28

**Authors:** Brenda Mello Franco, Tauana Fernanda Jantsch Voigt, Juliana Felipetto Cargnelutti, Sabrina Frantz Lütke, Cristiano Rodrigo Bohn Rhoden, Marcos Leandro Silva Oliveira, Luis Felipe Oliveira Silva, Guilherme Luiz Dotto

**Affiliations:** † 28118 Universidade Federal de Santa Maria, Research Group on Adsorptive and Catalytic Process Engineering (ENGEPAC), Av. Roraima, 1000-7, Santa Maria 97105-900, Brazil; ‡ Universidade Federal de Santa Maria, Graduate Program in Veterinary Medicine, Av. Roraima, 1000, Santa Maria 97105-900, Brazil; § Universidade Federal de Santa Maria, Department of Preventive Veterinary Medicine, Av. Roraima, 1000, Santa Maria 97105-900, Brazil; ∥ Universidade Federal de Santa Maria, Chemistry Department, Av. Roraima, 1000, Santa Maria 97105-900, Brazil; ⊥ 42510 Universidade Franciscana, Laboratory of Nanostructured Magnetic Materials (LaMMaN), 97010-032 Santa Maria, RS, Brazil; # 27951 Corporacion Universitaria de la Costa, Calle 58 #55−66, Barranquilla 080003, Atlántico, Colombia

## Abstract

Silica was obtained from rice husk, a low-cost and abundant agricultural waste, and chemically modified with three phthaloyl diamide ligand isomers, aiming to develop new materials with adsorptive and antibacterial potential. The prepared adsorbents were fully characterized, confirming the successful functionalization. The isomeric structure of the ligands significantly influenced both the uniformity and extent of functionalization and the Ag^+^ adsorption efficiency. The Ag^+^ adsorption was favored at pH 5.0 and room temperature, and the adsorption rate was very fast, reaching equilibrium in about 15 min. The maximum adsorption capacity was 19.94 mg g^–1^. Antibacterial tests demonstrated that the Ag^+^-loaded adsorbent exhibited marked antibacterial activity against *Escherichia coli*, highlighting the dual functionality of the synthesized material. These results underscore the potential of phthaloyl-modified rice husk-derived silica as a novel and effective material for integrated Ag^+^ removal and antibacterial protection.

## Graphical abstract

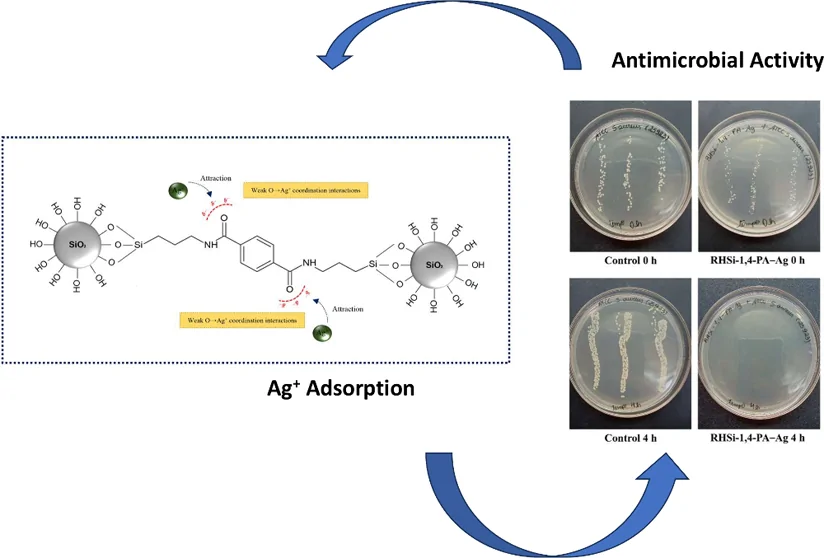

## 1 Introduction

The contamination of aquatic environments by heavy metals is a globally relevant problem, mainly due to their persistence, nonbiodegradability, bioaccumulation, and toxicity. These characteristics contribute to the gradual buildup of heavy metals in ecological systems, posing serious risks to aquatic organisms and, indirectly, to human health through bioaccumulation and biomagnification in the food chain.^1–3^ Among the various heavy metals, silver (Ag) has garnered growing attention due to its increasing use across industrial sectors such as photography, catalysts, conductors, electrical devices, health care products, clothing, cosmetics, water filters, and paints, among others.^4,5^ Thus, with the increased manufacturing and use of Ag, there has been a growing anthropogenic input of Ag into the environment. As a result, Ag contamination in wastewater has become a critical issue, especially given its toxicity to plant species, marine and soil organisms, animals, and human beings.^4^

Since untreated silver-ion-containing wastewater can severely damage the environment and human health, with impacts that vary depending on the type of wastewater and production process,^6^ the search for efficient, selective, and economically viable remediation strategies has intensified. In this context, adsorption stands out as a highly promising approach due to its operational simplicity, versatile design, economic feasibility, lack of sludge formation, and potential for adsorbent regeneration and reuse.^2,6,7^ However, achieving high efficiency in Ag^+^ removal depends heavily on the adsorbent’s physicochemical properties, including surface chemistry, porosity, and interaction capacity with the target ion. In this context, the functionalization of the adsorbent with organic ligands containing specific functional groups represents a promising alternative. The incorporation of suitable functional groups at the adsorbent’s surface, such as amides, amines, or carbonyls, can improve its adsorption performance through the formation of stable complexes with the Ag^+^ ions^8–10^

In recent years, the valorization of agricultural waste has emerged as a sustainable and strategic alternative for developing adsorbent materials. Agricultural residues are abundant, renewable, and low-cost, and their use as precursor materials for the production of adsorbents aligns with the principles of the circular economy.^11–13^ In particular, rice husk, a byproduct of rice processing, contains a significant proportion of silica (approximately 15 to 20%), which can be obtained through controlled thermal treatment followed by acid leaching, yielding high-purity amorphous silica.^14–16^ The resulting rice husk silica has a porous structure, and its surface silanol groups (Si–OH) make it a promising support for functionalization and adsorption applications.^17,18^

Building upon this context, the functionalization of rice husk-derived silica with organic ligands offers an attractive route to tailor its surface chemistry for specific contaminant removal. This work proposes the synthesis, characterization, and application of rice husk-derived silica functionalized with phthaloyl diamide (PA) ligands, derived from three different phthaloyl chloride isomers (1,2-, 1,3-, and 1,4-PACl). The obtained adsorbents were characterized regarding their textural, morphological, chemical, and thermal properties, and their adsorption performance for Ag^+^ was assessed under varying experimental conditions. The pH effect, the adsorption kinetics, equilibrium, and thermodynamics were investigated, as well as the regeneration and reuse potential. The antibacterial activity of the Ag^+^–loaded adsorbent was also investigated.

A particularly novel aspect of the present study is the exploration of positional isomerism of the attached chelating ligands, investigating the influence on surface functionalization efficiency and adsorption performance. The evaluated isomers differ in the relative positions of the functional groups on the aromatic ring, which can affect conformation, steric hindrance, accessibility of the functional groups, and, ultimately, their interactions with Ag^+^ ions. By systematically comparing these isomers, the present work contributes to a deeper understanding of structure–performance relationships in functionalized adsorbents, an aspect that is largely overlooked in the literature. Moreover, the study goes beyond adsorption performance and investigates the antibacterial activity of the Ag^+^-loaded materials against *Staphylococcus aureus* and *Escherichia coli*, thereby assessing the materials’ dual functionality. By integrating sustainable material sourcing, functionalization, and comprehensive performance evaluation, this study advances efficient water treatment technologies.

## 2 Materials and Methods

### 2.1 Materials and Reagents

The rice husk (*Oryza sativa*) used in this work for silica production was provided by a local rice processing industry. (3-Aminopropyl)­triethoxysilane (APTS, purity of 99%), phthaloyl chloride (1,2-PACl, purity of 90%), isophthaloyl dichloride (1,3-PACl, purity of 99%), and terephthaloyl chloride (1,4-PACl, purity of 99%) were acquired from Sigma-Aldrich. The organic solvents toluene, triethylamine, and dichloromethane were acquired from Neon (Brazil) and were all of analytical grade. Toluene and triethylamine were dried with metallic sodium (Dinâmica, Brazil) before use. Other reagents were used as received. Hydrochloric acid (HCl), sulfuric acid (H_2_SO_4_), sodium hydroxide (NaOH), and sodium chloride (NaCl), also of analytical grade and obtained from Neon (Brazil), were employed for silica purification, pH adjustment, and point of zero charge (pH_PZC_) determination. Silver solutions used in the batch adsorption experiments were prepared from Ag_2_SO_4_ (Vetec, Brazil).

### 2.2 Preparation of Rice Husk Silica (RHSi)

The rice husk was initially washed with deionized water, dried in an oven (Solab, SL–101, Brazil) at 50 °C for 24 h, ground, and sieved. Then, the pretreated rice husk was calcined in a muffle furnace (Magnus, model 06, Brazil) at 600 °C for 6 h to remove organic matter. The resulting ash was subjected to conventional acid leaching with a 10% (w/w) hydrochloric acid (HCl) solution, stirred at 80 °C for 2 h in a reflux system. This purification step was carried out to remove metallic impurities [15,16]. After the acid treatment, the material was washed with deionized water until neutral pH, filtered, and dried at 100 °C for 24 h in an oven (Solab, SL–101, Brazil). The dried material was macerated and subsequently recalcined (Magnus, model 06, Brazil) at 700 °C with a heating rate of 5 °C min^–1^.^16^ Due to the low silica content obtained in the first purification step, the entire purification and recalcination process was repeated to improve the purity of the final material. The resulting material was named RHSi.

### 2.3 Synthesis of the Ligands and RHSi Functionalization

The synthesis of the ligands based on phthaloyl diamide and the functionalization of the RHSi were carried out according to Hu et al.^19^ The ligands were prepared from phthaloyl chloride (1,2-PACl), isophthaloyl chloride (1,3-PACl), and terephthaloyl chloride (1,4-PACl) (Figure 1). For the synthesis, 10 mmol of each chloride (1.53 mL of 1,2-PACl, 2.04 g of 1,3-PACl, and 2.05 g of 1,4-PACl) was initially dissolved in 25 mL of dry toluene. Then, the dropwise addition of a solution of 4.9 mL of APTS and 14 mL of dry triethylamine in 20 mL of dry toluene was carried out under constant slow stirring provided by a magnetic stirrer. For 1,3-PACl, the addition was carried out at 0 °C, then allowed to warm to room temperature. After the addition, the mixtures were stirred at room temperature overnight. For the synthesis of the phthaloyl ligand from 1,2-PACl, the reaction was carried out under an inert atmosphere. After the reaction times, the mixtures were vacuum-filtered, and the solvent was removed using a rotary evaporator.

**Figure 1:**
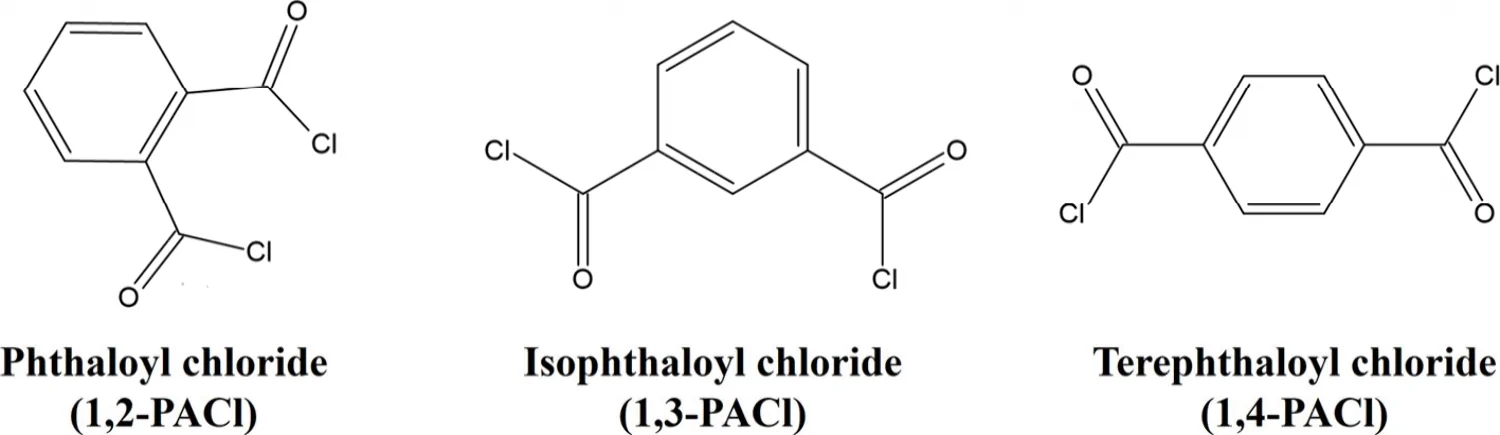
Structural formula of phthaloyl chloride, isophthaloyl chloride, and terephtaloyl chloride.

For the surface functionalization, RHSi was first dried in a vacuum oven (Laborglas, LGI-VO-6030B, Brazil) at 150 °C for 24 h to remove adsorbed moisture. Subsequently, 1 g of the dried RHSi was dispersed in 60 mL of dry toluene and held for 20 min. After this time, 0.46 g of the previously synthesized ligands and 1.2 mL of triethylamine were added all at once to the suspension, and the mixture was kept under reflux for 24 h. Upon completion, the functionalized adsorbents were vacuum-filtered and washed with toluene. Finally, the materials were washed with a Soxhlet apparatus using dichloromethane for 12 h to remove unreacted silane molecules and dried at 100 °C for 8 h in a vacuum oven (Laborglas, LGI-VO-6030B, Brazil). The functionalized adsorbents were named RHSi-1,2-PA, RHSi-1,3-PA, and RHSi-1,4-PA, according to the starting reagent, 1,2-PACl, 1,3-PACl, and 1,4-PACl, respectively. Figure 2 shows a schematic representation of the preparation of the materials, using RHSi-1,4-PA as an example.

**Figure 2:**
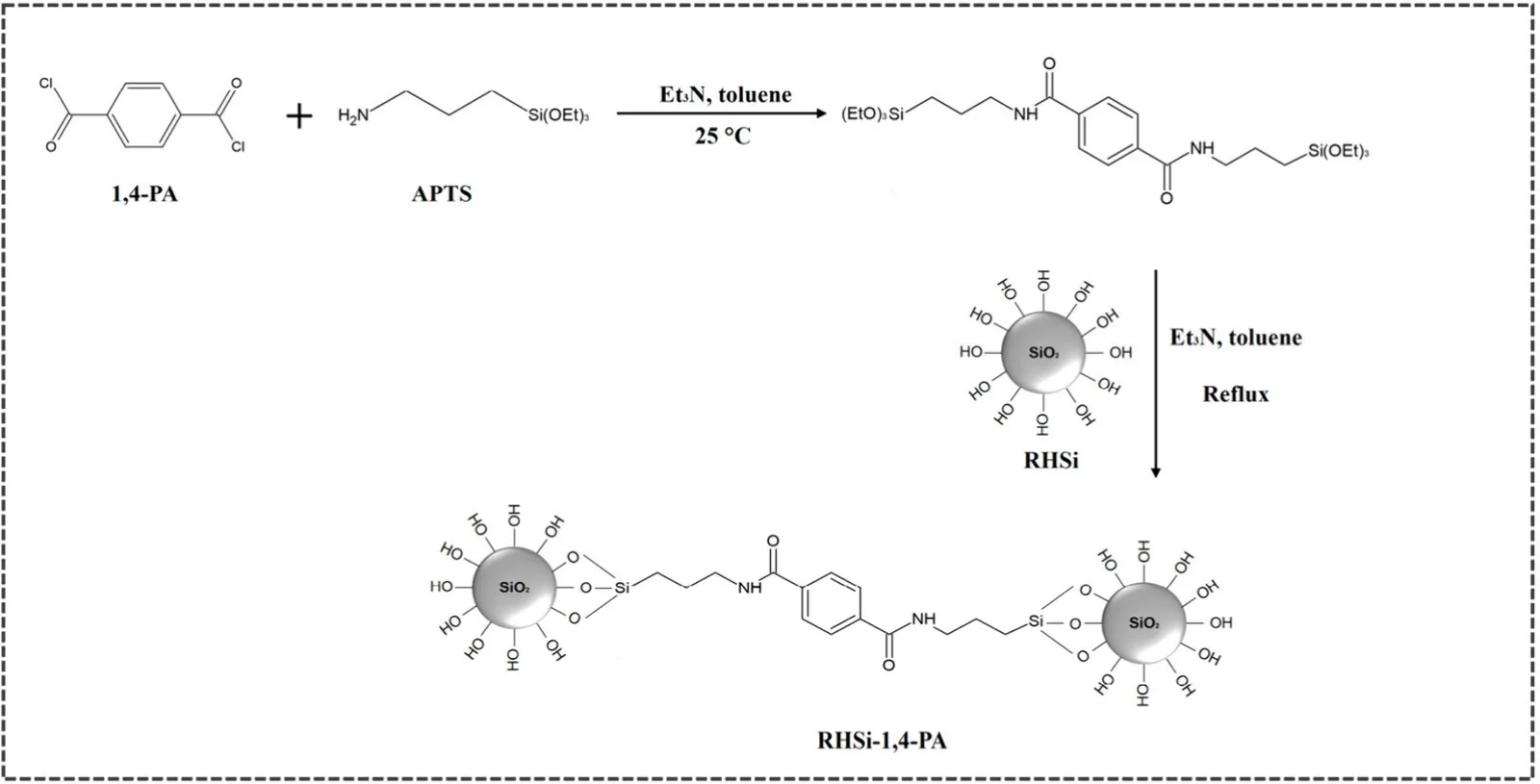
Schematic representation of the synthesis of the functionalized rice husk-derived silica (using RHSi-1,4-PA as an example).

### 2.4 Characterization of Rice Husk-Derived Silica and Functionalized Rice Husk-Derived Silica

To confirm the success of the synthesis of rice husk silica and the functionalization of this material by the phthaloyl diamide ligands, several characterization analyses were carried out. The silica content in the RHSi sample was estimated by X-ray fluorescence (XRF), and its surface morphology and elemental composition were analyzed by scanning electron microscopy (SEM) and energy-dispersive X-ray spectroscopy (EDS), respectively.

The surface functional groups of the RHSi and the modifications resulting from functionalization were identified by Fourier transform infrared (FTIR) spectroscopy. Powder X-ray diffraction (XRD) was used to verify the crystallinity of the RHSi and functionalized adsorbents. The thermal profile was obtained by thermogravimetric analysis (TGA). Textural properties, i.e., surface area, total pore volume, and average pore size, were determined based on N_2_ adsorption/desorption isotherms, using Brunauer–Emmett–Teller (BET) and Barrett–Joyner–Halenda (BJH) methods. The point of zero charge (pH_PZC_) was obtained to determine the net surface charge of RHSi and functionalized adsorbents at a given pH.

More information on the conditions for the characterization analyses can be found in the Supporting Information (section S.1).

### 2.5 Ag^+^ Adsorption Experiments

Batch adsorption experiments were conducted to evaluate the performance of RHSi and functionalized silica adsorbents for the removal of Ag^+^ ions from synthetic aqueous solutions. Aqueous Ag^+^ solutions were prepared from silver sulfate (Ag_2_SO_4_). In each experiment, 20 mL of the Ag^+^ aqueous solution was mixed with 20 mg of the adsorbent (adsorbent dosage of 1 g L^–1^) in 125 mL Erlenmeyer flasks and agitated in a shaker incubator (Solab, SL 222, Brazil) at 150 rpm.

To initially investigate the effect of pH on Ag^+^ adsorption using RHSi, RHSi-1,2-PA, RHSi-1,3-PA, and RHSi-1,4-PA, tests were performed with an initial Ag^+^ concentration of 20 mg L^–1^ and pH values ranging from 2 to 6. The desired pH values were adjusted by adding NaOH or H_2_SO_4_ solutions (0.1 mol L^–1^), and the pH range of 2–6 was chosen to avoid silver precipitation in the solution. The pH effect tests were conducted at 30 °C and a contact time of 24 h.

After determining the most adequate pH condition for Ag^+^ adsorption, kinetic studies were carried out with initial Ag^+^ concentrations of 20, 50, 100, 150, and 200 mg L^–1^, varying the contact time at set intervals (0, 2, 5, 10, 15, 20, 30, 40, 60, 90, 120, 180, and 240 min). Lastly, equilibrium curves were obtained at 30, 40, 50, and 60 °C, using the same initial Ag^+^ concentrations as in the kinetic adsorption experiments. The solutions were maintained in the shaker incubator for the time required until equilibrium was reached.

The experiments were performed in triplicate, and the average values were reported. Blank tests were also performed under the same conditions but without adsorbents. After each adsorption test, the samples were centrifuged (LGI Scientific, LGI-DLC-802B, Brazil), and the remaining Ag^+^ concentration in the liquid phase was determined by atomic absorption spectroscopy (AAS). AAS analysis was carried out using a flame atomic absorption spectrometer (Agilent Technologies, 240FS AA, USA) equipped with a hollow cathode lamp for silver, operated at 328.1 nm, using an air-acetylene flame and external calibration with Ag^+^ standards ranging from 2.5 to 10.0 mg L^–1^. The performance of the adsorbents was assessed by determining the adsorption capacity at time *t* (*q*_*t*_), equilibrium adsorption capacity (*q*_*e*_), and removal percentage (*R*, %) according to eqs 1, 2, and 3, respectively:

1 $$q_{t}=\frac{V\left(C_{0}-C_{t}\right)}{m}$$

2 $$q_{e}=\frac{V\left(C_{0}-C_{e}\right)}{m}$$

3 $$R\left(\%\right)=\frac{\left(C_{0}-C_{e}\right)}{C_{0}}100$$

where *C*_0_, *C*_*t*_, and *C*_*e*_ (mg L^–1^) are the adsorbate concentrations in the liquid phase at the beginning, time *t*, and equilibrium, respectively; *m* (g) is the adsorbent mass; and *V* (L) is the volume of the solution.

### 2.6 Kinetics, Equilibrium, and Thermodynamics Calculation

To further understand silver adsorption behavior, adsorption kinetics and equilibrium models were used, and the thermodynamic parameters were estimated. Details can be seen in the Supporting Information (Section S.2).

### 2.7 Regeneration and Reuse Experiments

The reusability of the adsorbent was evaluated through regeneration experiments using different mineral acids. After loading the adsorbent with Ag^+^ ions, the solid phase was separated by filtration, thoroughly washed with deionized water, and subsequently treated with 50 mL of regenerating solution (HCl, H_2_SO_4_, or HNO_3_) at concentrations of 0.5 and 1.0 mol L^–1^. During the regeneration step, the mixture was stirred at 150 rpm (Solab, SL 222, Brazil) for 120 min at room temperature to promote desorption. After that, the material was filtered, washed with deionized water until neutral pH, dried at 60 °C, and then reused in a subsequent adsorption cycle. This adsorption–desorption–reuse procedure was repeated to assess the material’s regeneration efficiency and stability.

### 2.8 Antibacterial Activity Investigation

The antibacterial potential of the Ag^+^-loaded adsorbent was evaluated against *Staphylococcus aureus* (ATCC 25923) and *Escherichia coli* (ATCC 25922) using a broth microdilution assay, following the protocol established by the Laboratory of Bacteriology and Antibiotic Resistance (LABAC/UFSM). Bacterial suspensions were prepared in Mueller–Hinton (MH) broth and adjusted to a 0.5 McFarland standard, corresponding to approximately 10^8^ CFU mL^–1^. Each sample (RHSi-1,4-PA and RHSi-1,4-PA-Ag^+^) was tested at a concentration of 10 mg mL^–1^. Additionally, free Ag^+^ ions with an identical loading amount as the Ag^+^-loaded adsorbent (RHSi-1,4-PA-Ag^+^) were also evaluated against *Staphylococcus aureus* and *Escherichia coli*.

The suspensions were incubated at 37 °C for 4 h. Following incubation, aliquots were serially diluted and plated on MH agar, then incubated for 24 h at 37 °C. Colony counts were expressed as log_10_ CFU mL^–1^. Controls included a positive control (bacteria only) and a negative control (sterile MH broth; data not shown).

## 3 Results and Discussion

### 3.1 Rice Husk Silica and Functionalized Adsorbents Characterization

#### 3.1.1 Morphological and Physical Characterization

Figure 3 shows the micrographs of the RHSi adsorbent obtained by scanning electron microscopy (SEM) with magnifications of 1000× and 5000× and the energy-dispersive X-ray spectroscopy (EDS) spectra. From Figure 3a, it is possible to observe that the original rice husk silica consists of irregular particles of different sizes and shapes, with many smaller granules. Figure 3b shows that the material’s surface is quite uneven and rough, with some cavities. The EDS spectrum of the RHSi adsorbent (Figure 3c) reveals that the main elements are oxygen (O) and silicon (Si), as expected from the composition of the material (SiO_2_). Carbon (C) is present at lower levels, which may be due to the formation of char during the calcination step. Traces of potassium (K), calcium (Ca), and magnesium (Mg) can also be seen in the EDS spectrum of the RHSi adsorbent, which appear as impurities. However, the very small percentages of these elements indicate a high-purity material.

**Figure 3:**
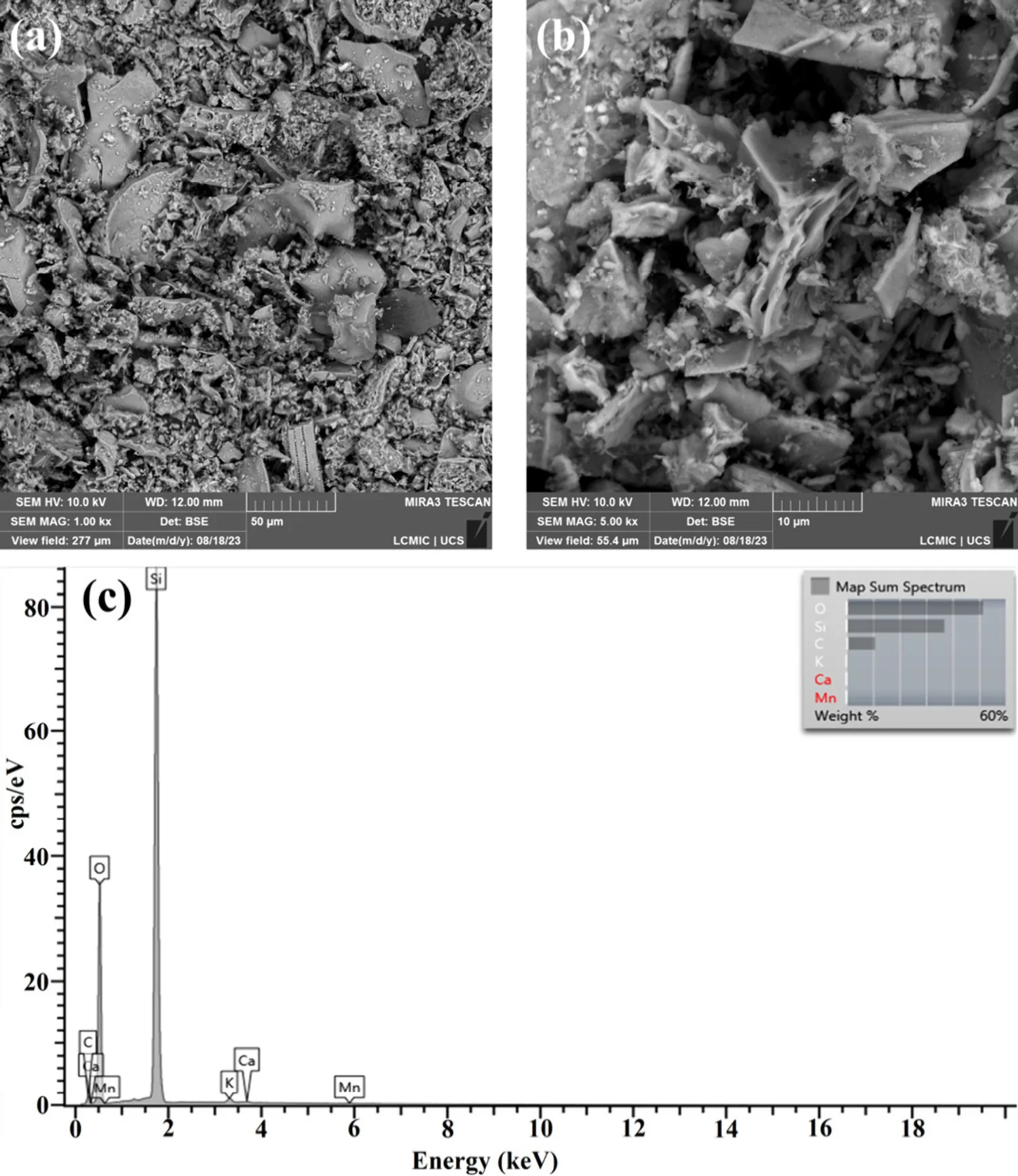
(a) SEM image of the RHSi adsorbent with magnification of 1000×, (b) SEM image of the RHSi adsorbent with magnification of 5000×, and (c) EDS spectrum of the RHSi adsorbent.

Figure 4 shows the micrographs of the rice husk silica after the functionalization. The functionalized adsorbents also exhibited irregular particles with rough surfaces and cavities, revealing no substantial change compared to the original rice husk silica, as expected. The SEM images of the RHSi-1,4-PA adsorbent (Figure 4e and f) show rougher particles with more cavities, compared to those of the other functionalized adsorbents. RHSi-1,2-PA and RHSi-1,3-PA adsorbents, on the other hand, exhibit less rough surfaces with fewer cavities, as evidenced by the SEM images in Figure 4 (a–d). However, higher magnification SEM images of the RHSi-1,2-PA and RHSi-1,3-PA adsorbents (Figure 4b and 4d) reveal the presence of numerous small pores that are not visible in the SEM image of the RHSi-1,4-PA adsorbent (Figure 4f).

**Figure 4:**
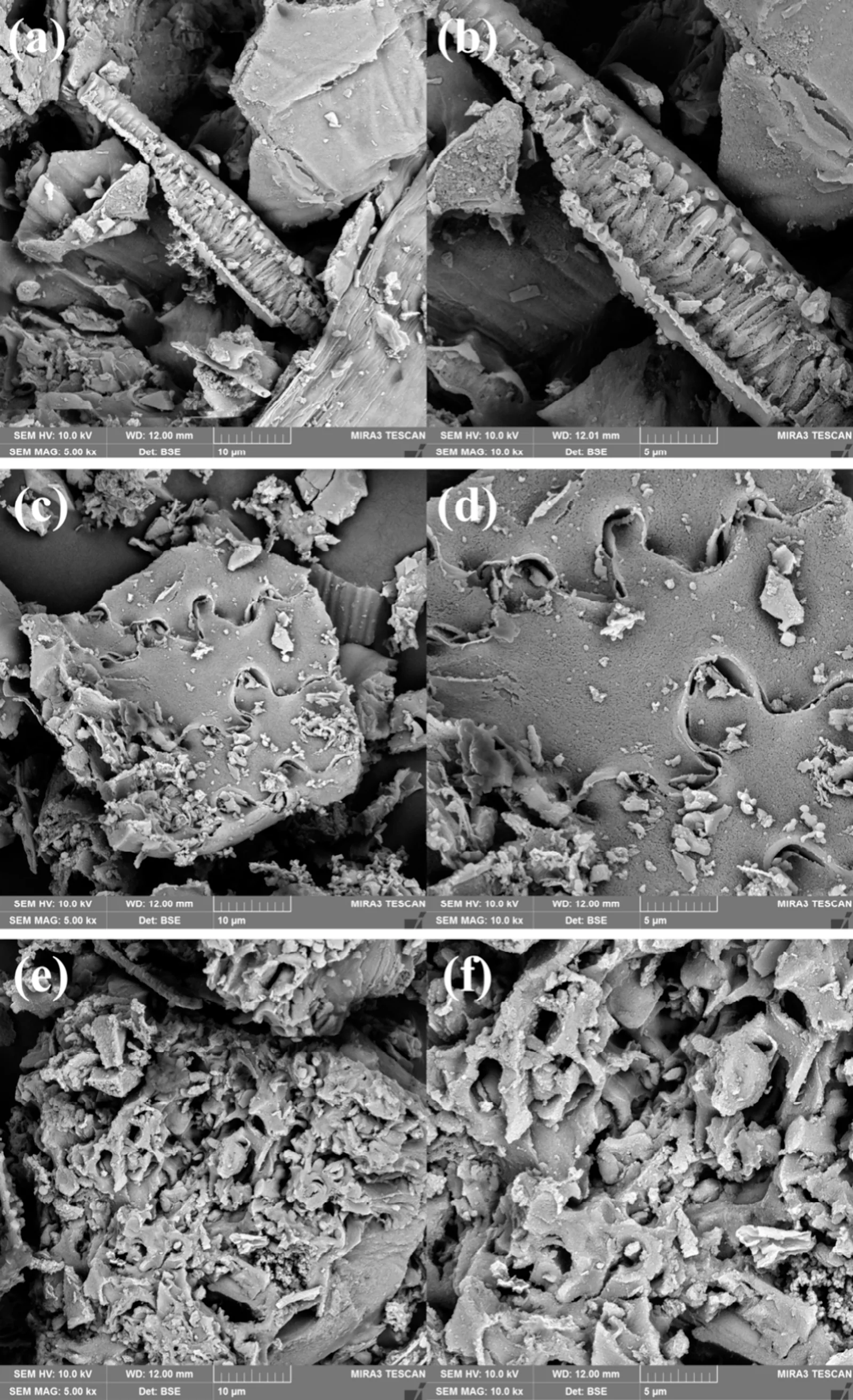
SEM images of (a) RHSi-1,2-PA with magnification of 5000×, (b) RHSi-1,2-PA with magnification of 10000×, (c) RHSi-1,3-PA with magnification of 5000×, (d) RHSi-1,3-PA with magnification of 10000×, (e) RHSi-1,4-PA with magnification of 5000×, and (f) RHSi-1,4-PA with magnification of 10000×.

In particular, the SEM images of the RHSi-1,2-PA adsorbent (Figure 4a and 4b) clearly show a cross-sectional area of a particle, positioned at the center of the image. The observed fiber-like morphology is attributed to the silica-rich structure naturally embedded in the inner epidermal layer of the rice husk. This fibrous framework, primarily composed of amorphous silica and cellulose, is known to originate from silica bodies that accumulate during plant growth.^20,21^ The presence of this structure supports the notion that the rice husk’s intrinsic microstructure is retained to some extent during functionalization and pyrolysis, which may contribute to the overall porosity and surface area of the resulting adsorbents.

The N_2_ adsorption/desorption isotherms and pore size distributions of the RHSi sample and functionalized adsorbents (RHSi-1,2-PA, RHSi-1,3-PA, and RHSi-1,4-PA) are presented in Figure 5. According to the IUPAC classification, the N_2_ adsorption curves (Figure 5a–d) can be classified as Type III, accompanied by a Type H3 hysteresis loop. This behavior is characteristic of mesoporous materials.^22^ Besides that, since adsorption/desorption curves obtained for the functionalized materials showed the same profile as for the original RHSi, it can be concluded that the functionalization did not modify the porous structure of the rice husk silica.

**Figure 5:**
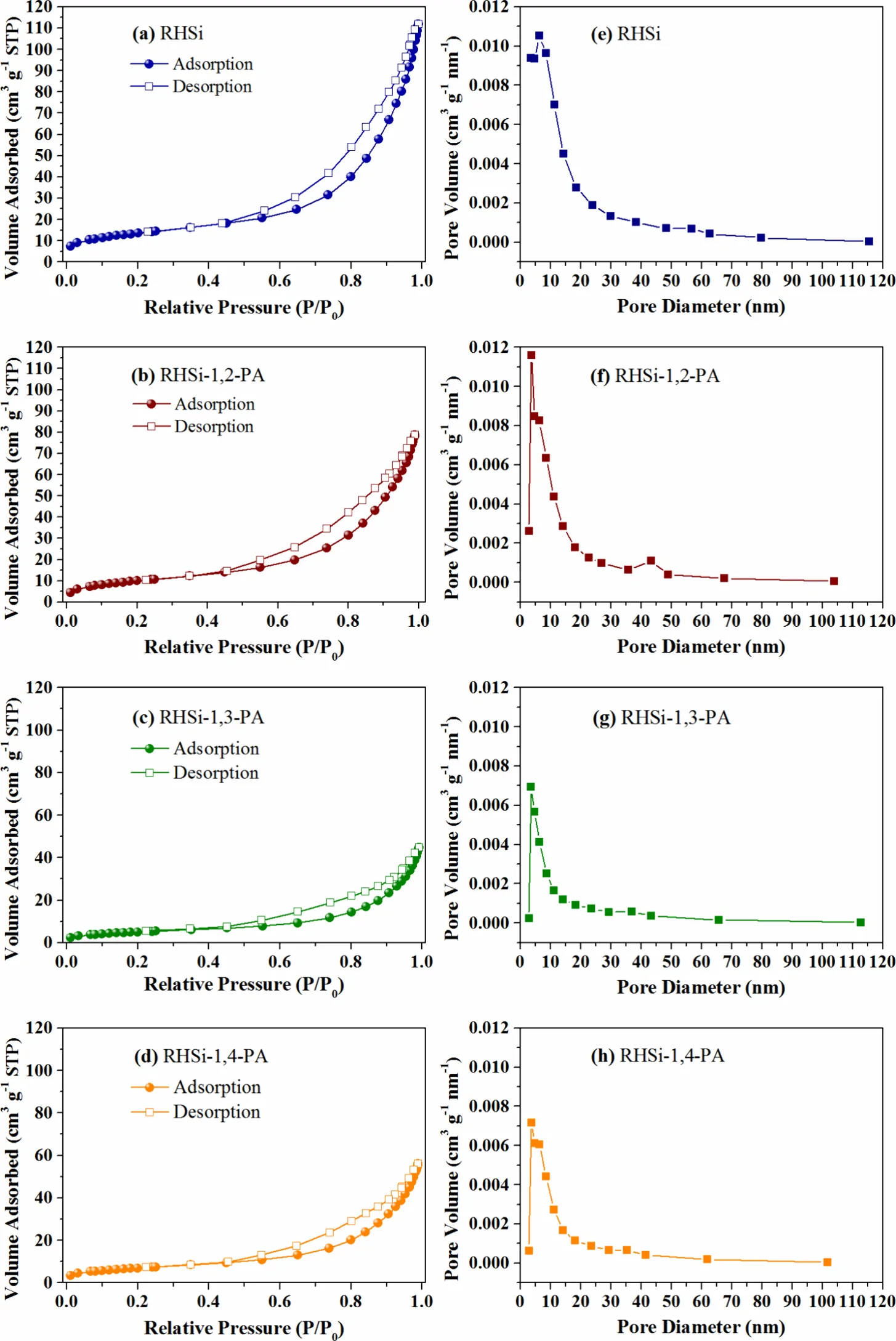
N_2_ adsorption–desorption isotherms (a–d) and pore size distribution calculated using the BJH method (e–h) for the synthesized adsorbents.

Figure 5e–h shows the pore size distribution obtained by the BJH method. RHSi sample (Figure 5e) exhibited a narrow pore size distribution with a peak centered at 6.3 nm (pore diameter of 12.6 nm), indicating mesoporous properties. For RHSi-1,3-PA (Figure 5g) and RHSi-1,4-PA (Figure 5h), it is possible to observe that the pore size distribution remained narrow, indicating homogeneous surface functionalization and the absence of pore blocking.^19^ However, peaks shift to lower values, i.e., 3.5 nm (pore diameter of 7.0 nm) for RHSi-1,3-PA and 3.6 nm (pore diameter of 7.2 nm) for RHSi-1,4-PA, indicating a decrease in pore size after the functionalization. For RHSi-1,2-PA, however, the pore size distribution (Figure 5f) indicated a peak centered at 3.7 nm (pore diameter of 7.4 nm) followed by a peak at 6.3 nm (pore diameter of 12.6 nm). Additionally, a broader peak around 43 nm is observed for the RHSi-1,2-PA sample. This larger pore radius is not typically related to mesoporosity but may result from interparticle voids or macropores formed during aggregation. These findings suggest that ligand functionalization did not occur homogeneously across the surface of the RHSi material. A possible explanation for this lies in the molecular structure of the 1,2-PACl-derived ligand. The proximity of the two acyl chloride groups in the ortho position can cause significant steric hindrance, known as the ortho-effect,^23^ making it more difficult for the ligand to evenly attach to all available silanol groups on the silica surface. As a result, some regions may remain less functionalized.

This interpretation is supported by the pore size distribution, which shows two distinct peaks, one resembling that of unmodified RHSi and another shifted, indicating an uneven modification process.

The textural properties, i.e., the specific surface area, total pore volume, mesopore volume, and average pore diameter, obtained for the synthesized adsorbents are shown in Table 1. RHSi presented specific surface area, total pore volume, and mesopore volume values of 49.13 m^2^ g^–1^, 0.195 cm^3^ g^–1^, and 0.058 cm^3^ g^–1^, respectively. Table 1 also shows that the RHSi presented a purity content of 97.9% (calculated by XRF measurements). Many studies in the literature have also reported similar textural properties and purity levels for silica obtained from rice husk. Nassar, Ahmed, and Raya,^24^ for example, produced and purified silica from rice husk by acid leaching followed by extraction with a 2.0 mol L^–1^ sodium hydroxide solution, and the obtained material showed a specific surface area and total pore volume of 59.28 m^2^ g^–1^ and 0.176 cm^3^ g^–1^, respectively, and a purity of 92–95%. Similarly, Chun et al.,^25^ in their study on the synthesis of silica from rice husk and purification via acid leaching, obtained specific surface areas and total pore volumes of 88 m^2^ g^–1^ and 0.18 cm^3^ g^–1^, respectively, and reported silica purity levels of 98–99%. Peres et al.^16^ also synthesized silica from rice husk and purified it via acid leaching, reporting specific surface area and total pore volume of 71.97 m^2^ g^–1^ and 0.2 cm^3^ g^–1^, respectively, and a purity of 97.8%. Therefore, the specific surface area, total pore volume, and purity content values obtained for the rice husk silica produced in the present work are within the expected range reported in the literature, confirming its adequate quality for subsequent functionalization and application in adsorption processes.

**Table 1:** Textural Properties (from N_2_ Physisorption) and Elemental Purity (from XRF) of Synthesized Adsorbents

Property	RHSi	RHSi-1,2-PA	RHSi-1,3-PA	RHSi-1,4-PA
Specific surface area (m^2^ g^–1^)	49.1 ± 2.3	37.2 ± 0.7	18.64 ± 1.6	25.5 ± 2.2
Total pore volume (cm^3^ g^–1^)	0.195 ± 0.022	0.123 ± 0.012	0.070 ± 0.009	0.087 ± 0.010
Mesopore volume (cm^3^ g^–1^)	0.058 ± 0.010	0.051 ± 0.008	0.025 ± 0.005	0.032 ± 0.008
Average pore diameter (nm)	10.43 ± 0.63	8.95 ± 0.65	9.72 ± 0.23	9.60 ± 0.15
Purity (%)	97.9 ± 0.1	–	–	–

For the functionalized adsorbents, a decrease in the specific surface area, total pore volume, and mesopore volume values was observed. This behavior can be attributed to the relatively large molecular size of the synthesized ligands, which tend to occupy the internal space of the porous network in rice husk silica. The extent of these reductions, however, may vary depending on the efficiency and uniformity of the functionalization. When functionalization is more homogeneous, a larger portion of the surface becomes blocked, resulting in a greater reduction in textural properties. In contrast, partial or uneven coverage leaves some regions accessible, leading to milder changes, as observed with the RHSi-1,2-PA adsorbent. Therefore, the differences observed among the materials in this study may be closely related to the distribution and degree of surface modification. These observations reinforce the notion that the isomeric structure of the ligands directly influences the efficiency and distribution of functionalization, ultimately affecting the textural properties of the materials.

In general, decreases in specific surface area and total pore volume are undesirable for adsorption applications, as they limit the number of active sites available for interaction with the adsorbate. Although RHSi-1,2-PA, RHSi-1,3-PA, and RHSi-1,4-PA exhibited inferior textural properties compared to unmodified RHSi, the presence of specific functional groups on their surfaces can compensate for these losses by enabling more effective interactions with Ag^+^ ions, thereby enhancing adsorption efficiency. These aspects will be discussed in greater detail in the following sections.

Lastly, as shown in Table 1, the average pore diameters confirm that all synthesized adsorbents are mesoporous, as they fall within the 2–50 nm range. A reduction in pore diameter was also observed after functionalization, which is due to the ligands occupying internal voids in the silica structure. A similar trend was reported by Hu et al.^19^ for KIT-6 silica functionalized with the same ligands used in the present study.

X-ray diffraction (XRD) analysis was carried out to confirm the amorphous nature of the RHSi adsorbent. In Figure 6, a broad, low-intensity peak centered at 2θ = 22° is observed, confirming that the obtained material has an amorphous structure. The amorphous nature observed for the RHSi adsorbent is consistent with the literature, which reports that crystalline SiO_2_ forms at temperatures above 700 °C, which is higher than the temperature used in the present work.^20,26,27^ The patterns obtained for the functionalized adsorbents (Figure 6) did not show relevant differences. RHSi-1,2-PA, RHSi-1,3-PA, and RHSi-1,4-PA, as shown in the red, blue, and orange lines, respectively, in Figure 6, also showed a single broad peak at 2θ = 22°. This finding indicates that functionalizing the RHSi adsorbent with the synthesized ligands did not alter its structure, preserving its amorphous nature.

**Figure 6:**
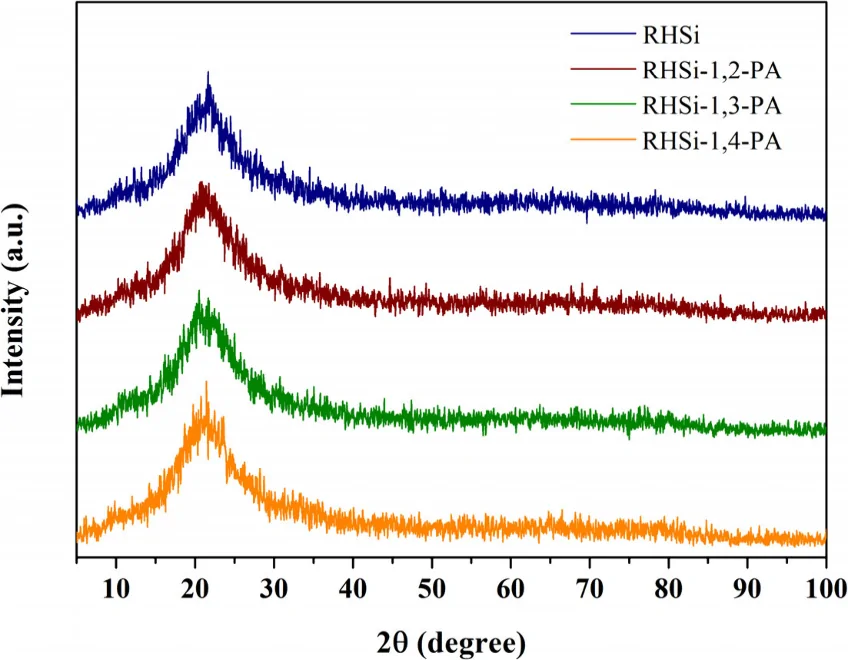
XRD patterns of RHSi and functionalized adsorbents.

#### 3.1.2 Chemical and Thermal Characterization

To investigate the functional groups on the surface of the RHSi and functionalized adsorbents, as well as to confirm the success of the functionalization through the covalent bonding of the synthesized ligands to silica, FTIR analysis was performed. First, in the FTIR spectra of the RHSi adsorbent (blue line in Figure 7), characteristic bands of silicon dioxide (SiO_2_) are observed at approximately 1100, 800, and 460 cm^–1^. The band at approximately 1100 cm^–1^ refers to the Si–O–Si asymmetric stretching vibration,^28^ symmetrical stretching vibration of Si–O–Si can be seen at around 800 cm^–1^, and the bending vibration of O–Si–O can be observed at around 460 cm^–1^.^29,30^ In addition, the vibrational band located at around 3450 cm^–1^ can be ascribed to the stretching of O–H and Si–OH bonds, commonly attributed to isolated silanol groups on the silica surface,^28^ and the band at around 1640 cm^–1^ is due to the stretching of the H–O–H bonds.^31^

**Figure 7:**
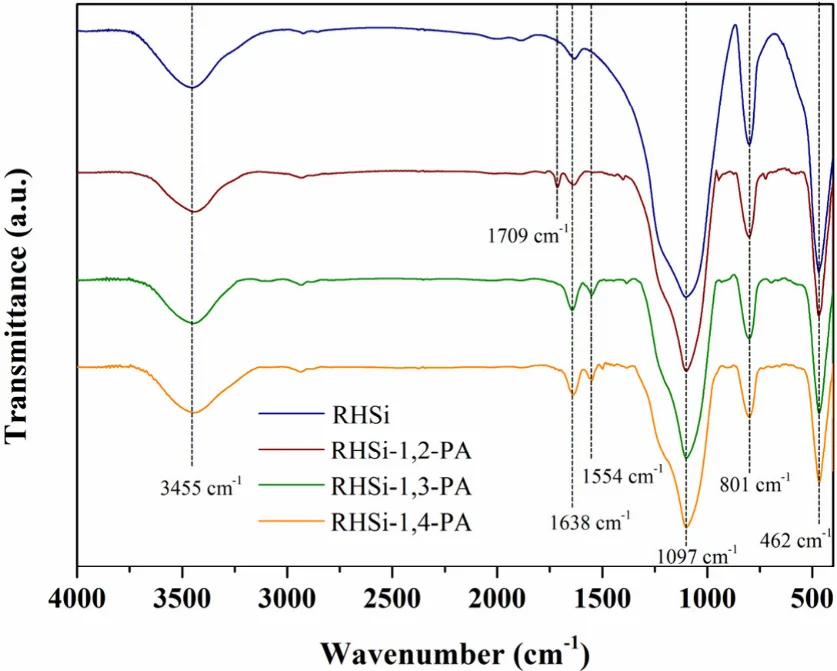
FTIR spectra of RHSi and functionalized adsorbents.

The FTIR spectra recorded for the functionalized adsorbents are also shown in Figure 7 (red, blue, and orange lines for RHSi-1,2-PA, RHSi-1,3-PA, and RHSi-1,4-PA adsorbents, respectively). It is important to initially mention that the characteristic silicon dioxide bands observed for the nonfunctionalized material (at around 1100, 800, and 460 cm^–1^) remained without any major change after the functionalization. Notwithstanding, the spectra obtained for the functionalized adsorbent exhibited new vibrational bands between 1700 and 1550 cm^–1^. The stretching vibration of the C = O bonds from the ligands can be confirmed by the emergence of the band around 1700 cm^–1^ or by the subtle increase of the band around 1640 cm^–1^. In addition, the band observed at around 1550 cm^–1^ for the functionalized adsorbents can be ascribed to the N–H bend in amine. Analogous results were found by Hu et al.^19^ These data confirm the success of the RHSi functionalization, with the covalent attachment of the ligands.

The TGA/DTG curves obtained for RHSi and functionalized adsorbents are shown in Figure 8. The thermogram of the RHSi adsorbent (Figure 8a) shows a single region of mass loss, with a sharp onset at 50 °C. The DTG peak can be observed around 115 °C and is attributed to the removal of moisture from the material surface.^32^ The thermal profiles obtained for the functionalized adsorbents (Figure 8b–d) show a region of mass loss starting from around 400 °C (with a DTG peak at approximately 475 °C for the three functionalized adsorbents), which can be attributed to the degradation process of the ligands. According to Hu et al. [19], these findings suggest covalent attachment of the ligands to the RHSi surface, thus confirming the success of the functionalization.

**Figure 8:**
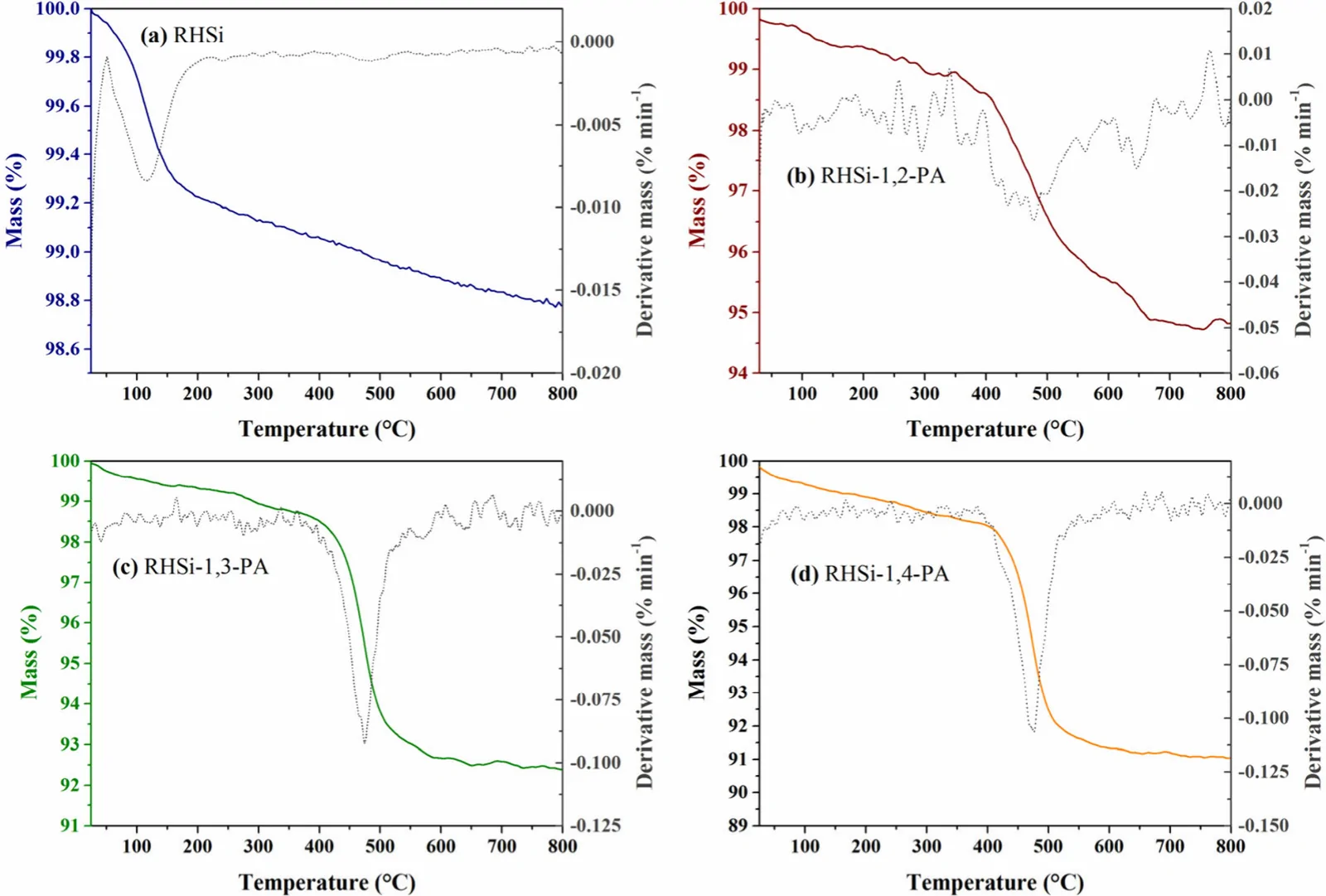
TGA/DTG curves of RHSi and functionalized adsorbents.

The thermal profile of the RHSi-1,2-PA material (Figure 8b) differs from the others, showing a less pronounced and more staged degradation event. This behavior can be explained by differences in ligand structure and/or binding modes. As already suggested, steric hindrance from the proximity of the two acyl chloride groups in the ortho position can lead to nonhomogeneous functionalization and variations in bonding strength. This fact can alter the thermal decomposition pathways, as observed in the thermal profile of RHSi-1,2-PA. In contrast, the 1,3- and 1,4-isomers possess more favorable molecular geometries, providing greater spatial separation between functional groups and increased conformational flexibility. These structural advantages may facilitate more efficient anchoring of the ligands to the silica surface.

### 3.2 Effect of pH on Ag^+^ Adsorption

The pH of the solution is a key parameter in adsorption processes, as it influences both the chemical speciation of the adsorbate and the surface charge behavior of the adsorbent. Figure 9 shows the effect of pH on the adsorption efficiency of Ag^+^ by RHSi and the functionalized adsorbents. All materials demonstrated increased Ag^+^ removal efficiency as the pH increased from 2.0 to 5.0. However, beyond pH 5.0, a slight decrease in the adsorption efficiency was observed for most adsorbents.

**Figure 9:**
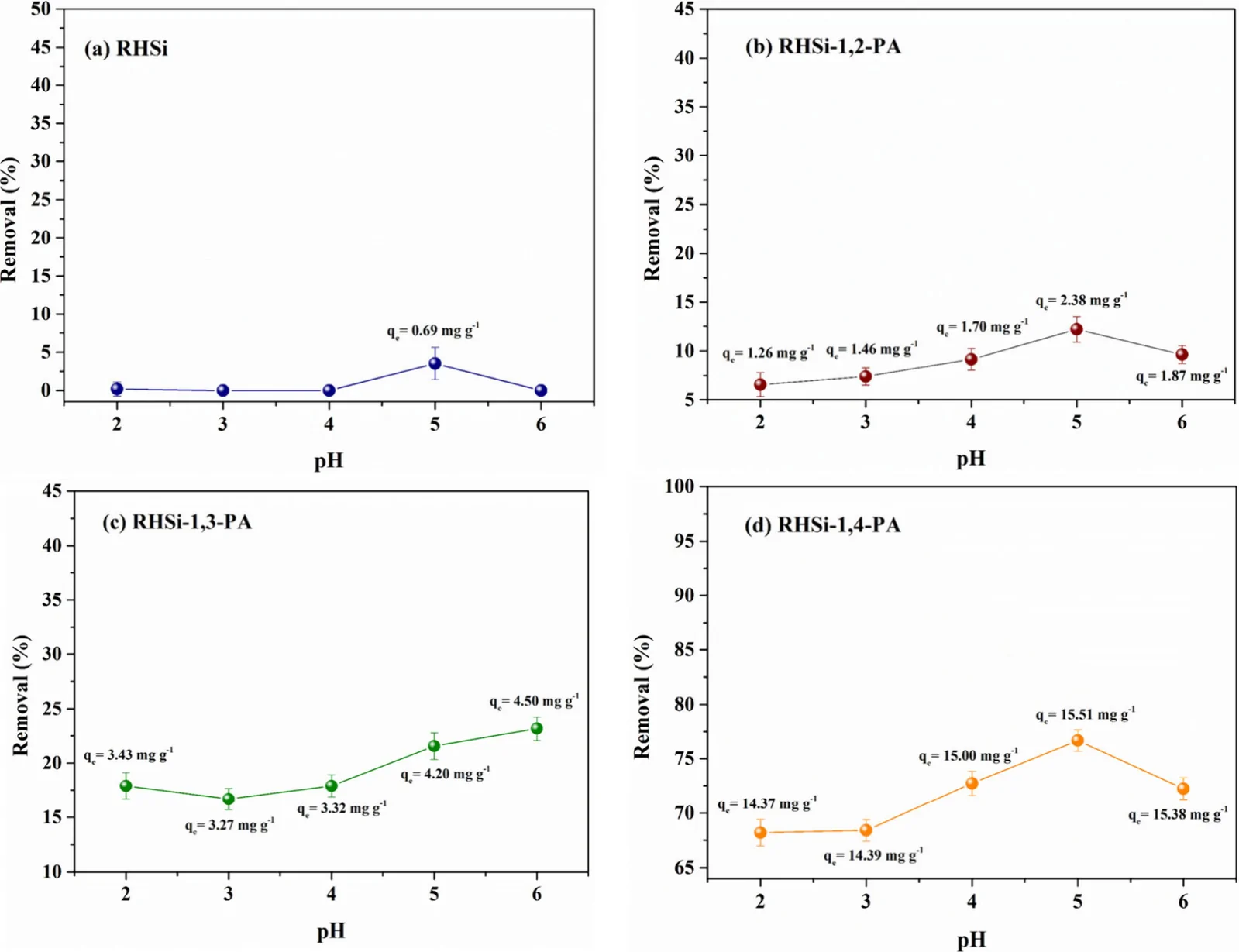
pH effect on Ag^+^ adsorption onto RHSi and functionalized adsorbents.

The point of zero charge (pH_PZC_) for the adsorbent materials was approximately 6.3, indicating that across the pH range tested (2 to 6), the adsorbent surfaces remained predominantly positively charged. Therefore, the classical explanation based on electrostatic attraction between negatively charged surfaces and Ag^+^ ions is not applicable in this case. Instead, the observed improvement in adsorption from pH 2 to 5 can be attributed to the reduced competition between protons (H^+^) and Ag^+^ ions for the available adsorption sites. At lower pH values, the high proton concentration can protonate surface functional groups, thereby inhibiting Ag^+^ coordination. As pH increases, protonation decreases, allowing functional groups to interact more effectively with Ag^+^ via specific interactions, such as complexation.

The onset of silver hydrolysis can partially explain the decline in adsorption efficiency beyond pH 5.0. At higher pH values, hydroxyls can compete with the adsorbent’s surface functional groups to form complexes with Ag^+^ ions, resulting in a slight decrease in removal efficiency.^33,34^ Thus, the optimum adsorption condition was found at pH 5.0, where proton interference is minimized, and silver remains predominantly in its free ionic form, allowing complexation with the electron-donor groups on the adsorbent surface.

Although the evaluated pH conditions (2–6) are below the point of zero charge of the adsorbents (pH_PZC_ = 6.3), which indicates a positive surface charge, the adsorbents still exhibit significant adsorption capacity. Even at very low pH values, the functionalized adsorbents, especially the RHSi-1,4-PA adsorbent, exhibited considerably high removal efficiencies. This behavior suggests that other adsorption mechanisms, beyond electrostatic interactions, may occur between the Ag^+^ ions and the functionalized surface, including specific chemical complexation. The presence of amide donor groups introduced by the phthaloyl diamide ligands can provide coordination sites that are effective at binding Ag^+^ ions. Several other studies in the literature also showed that ligands containing amide groups can promote the adsorption of metal cations by forming coordination complexes.^35–38^

All functionalized adsorbents (RHSi-1,2-PA, RHSi-1,3-PA, and RHSi-1,4-PA) outperformed unmodified RHSi in adsorbing Ag^+^. While nonfunctionalized RHSi relies primarily on silanol (Si–OH) groups that may interact weakly via hydrogen bonding, the introduction of the phthaloyl diamide ligands provides stronger donor atoms (O and N), enabling coordinative bonding with Ag^+^ ions.

Among the functionalized materials, RHSi-1,4-PA consistently outperformed the other adsorbents across the investigated pH range. As demonstrated by Hu et al.,^19^ the anchoring of the ligand derived from terephthaloyl chloride (carbonyl groups in para-positions) allows for more homogeneous coverage and enhanced ligand density. This characteristic can increase the accessibility of metal ions to active sites. In addition, the superior performance of RHSi-1,4-PA can be explained by the wider bite angle of the ligand derived from terephthaloyl chloride, which facilitates the simultaneous interaction of both donor atoms (oxygen and nitrogen) with the Ag^+^ ion, enhancing complexation efficiency. Therefore, the 1,4-isomer offers a structural advantage that enhances its complexation with Ag^+^ ions, resulting in superior adsorption performance.

### 3.3 Adsorption Kinetics

Considering the results found in the pH effect assays and the notably superior performance of the RHSi-1,4-PA adsorbent, the subsequent adsorption experiments were carried out at pH 5.0 and only for RHSi-1,4-PA. Figure 10 displays the kinetic profiles for the Ag^+^ adsorption onto RHSi-1,4-PA at different initial adsorbate concentrations (20–200 mg L^–1^). The obtained curves showed that the adsorption was very fast, reaching equilibrium in around 15 min for all evaluated Ag^+^ initial concentrations. This very fast adsorption kinetics is exceptionally favorable for large-scale applications, since large amounts of contaminants must be removed from effluents within short periods.^39^

**Figure 10:**
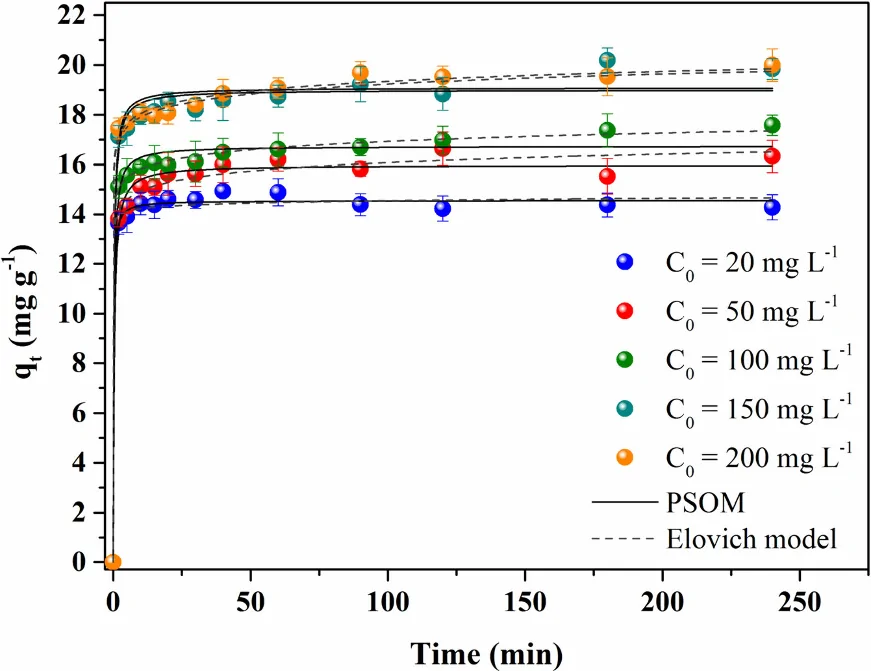
Kinetic curves of Ag^+^ adsorption onto RHSi-1,4-PA.

Similar kinetic behavior was observed over the evaluated adsorbate concentration (20 to 200 mg L^–1^), indicating that the process was independent of the initial adsorbate concentration. Additionally, increasing the initial adsorbate concentration from 20 to 150 mg L^–1^ resulted in a slight increase in adsorption capacity from around 14 to 20 mg g^–1^. However, no further increase in the adsorption capacity was observed at an initial concentration of 200 mg L^–1^, indicating that the available adsorption-active sites became saturated.

The time-dependent adsorption data were fitted to the PFOM, PSOM, and Elovich models, and the estimated parameters, along with the statistical metrics, are shown in Table 2. Both the PSOM and the Elovich model showed strong correlations with the experimental data, with *R* ^*2*^ values ranging from 0.989 to 0.999, *R* ^*2*^_adj_ values ranging from 0.987 to 0.999, *ARE* values ranging from 0.645 to 2.625, and *AIC* values ranging from −47.999 to −9.827. Therefore, both models suitably represented the Ag^+^ adsorption onto RHSi-1,4-PA.

**Table 2:** Kinetic Parameters for the Ag^+^ Adsorption onto RHSi-1,4-PA

	Initial Concentration (mg L^–1^)				
Model	20	50	100	150	200
PFOM					
*q*_1_ (mg g^–1^)	14.45	15.68	16.49	18.71	18.81
*k*_1_ (min^–1^)	1.422	1.026	1.218	1.207	1.287
*R* ^2^	0.996	0.984	0.986	0.972	0.982
*R*_adj_^2^	0.995	0.981	0.983	0.978	0.978
*ARE* (%)	1.27	2.67	2.61	2.76	3.18
*AIC*	–30.41	–10.13	–10.31	–3.76	–3.76
PSOM					
*q*_2_ (mg g^–1^)	14.55	15.97	16.72	19.00	19.09
*k*_2_ (g mg^–1^min^–1^)	0.484	0.165	0.219	0.183	0.199
*R* ^2^	0.997	0.993	0.992	0.989	0.989
*R*_adj_^2^	0.996	0.992	0.990	0.987	0.987
*ARE* (%)	1.24	1.96	2.06	2.39	2.62
*AIC*	–34.01	–21.20	–17.41	–10.30	–9.82
Elovich					
β (mg g^–1^min^–1^)	3.108	0.882	0.897	0.745	0.761
α (g mg^–1^)	5.2 × 10^42^	1.7 × 10^12^	1.7 × 10^13^	2.8 × 10^12^	7.16 × 10^12^
*R* ^2^	0.995	0.994	0.999	0.998	0.998
*R*_adj_^2^	0.994	0.992	0.999	0.997	0.998
*ARE* (%)	1.82	1.86	0.64	0.98	0.90
*AIC*	–26.40	–21.70	–47.99	–29.55	–34.22
*q*_*e*_ (mg g^–1^)	14.28	16.32	17.57	19.85	20.00

The high predictive ability demonstrated by the PSOM suggests that the adsorption of Ag^+^ onto RHSi-1,4-PA is mainly determined by site availability and interfacial interactions rather than external mass transfer alone.^40–43^ The estimated *q*_2_ values increased with increasing Ag^+^ initial concentration, corroborating the experimental data. In addition, for initial concentrations of 50–200 mg L^–1^, very similar *k*_2_ values were obtained (0.165–0.219 g mg^–1^ min^–1^), corroborating the experimental evidence that varying initial concentrations do not affect the adsorption rate. The Elovich model is an empirical kinetic model based on the assumption of adsorption on a heterogeneous surface and also describes the surface coverage adsorption mechanism.^44,45^ From Table 2, it is also evident that very similar β parameter values (0.745–0.897 mg g^–1^ min^–1^) were obtained for initial concentrations of 50–200 mg L^–1^, confirming a very small influence of the initial adsorbate concentration on the adsorption rate.

### 3.4 Equilibrium isotherms

Figure 11 shows the equilibrium adsorption isotherms of Ag^+^ onto RHSi-1,4-PA obtained at 303, 313, 323, and 333 K.

**Figure 11:**
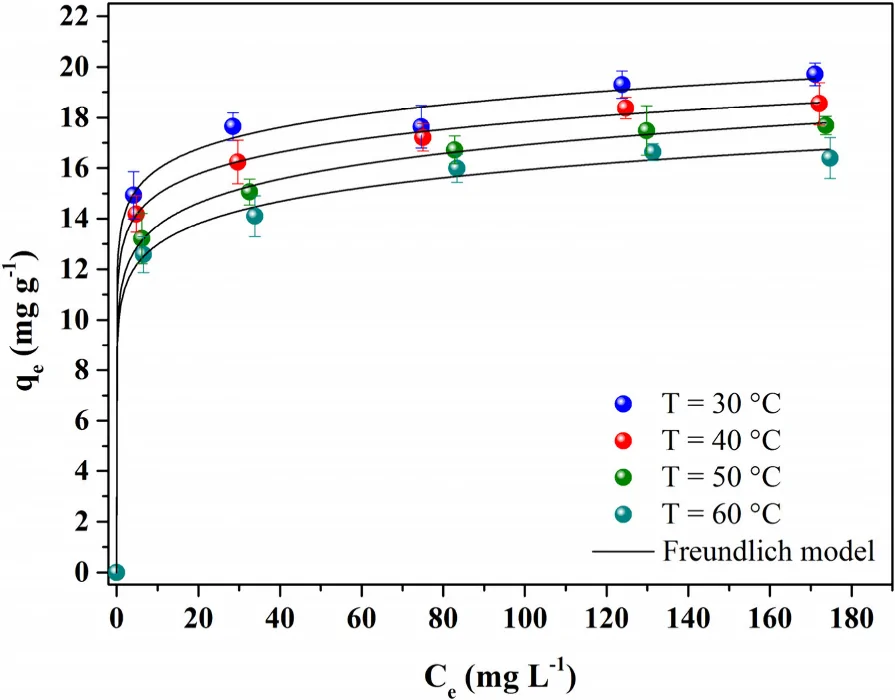
Equilibrium curves of the Ag^+^ adsorption onto RHSi-1,4-PA.

In all cases, the adsorption capacity increased with the equilibrium concentration (*C*_*e*_), while the maximum capacity decreased slightly with rising temperature, indicating less favorable adsorption at higher temperatures. According to the Giles classification, the profiles of the obtained equilibrium isotherms correspond to L-type, characteristic of systems with high adsorbate–surface affinity and limited solvent competition. This behavior suggests progressive occupation of active sites, as expected for amide groups covalently grafted onto the silica surface.^46,47^

The experimental data were fitted to Langmuir, Freundlich, and Sips models, and the corresponding parameters are summarized in Table 3. The Freundlich model provided the best statistical description at all temperatures (higher *R* ^2^_adj_ and lower *AIC* values), implying that the Ag^+^ uptake using RHSi-1,4-PA occurs on a heterogeneous surface with sites of different affinities, rather than on a uniform monolayer. Similar results were reported in the adsorption isotherm studies of various adsorbent-pollutant systems in the literature.^48–51^ In addition, 1/*n*_*F*_ < 1 further indicated favorable adsorption and decreasing site energy with increasing coverage.^47^

**Table 3:** Equilibrium Parameters for the Ag^+^ Adsorption onto RHSi-1,4-PA

	Temperature (K)			
Model	303	313	323	333
Langmuir				
*q*_*m*_ (mg g^–1^)	18.94	18.01	17.29	16.30
*K*_*L*_ (L mg^–1^)	0.848	0.721	0.478	0.468
*R* ^2^	0.*9*93	0.994	0.993	0.992
*R*_adj_^2^	0.989	0.990	0.989	0.987
*ARE*(%)	2.86	2.82	2.85	2.82
*AIC*	2.60	1.33	1.71	1.56
Freundlich				
*K*_*F*_ (mg g^–1^) (mg L^–1^)^−1/nF^	13.53	12.55	11.12	10.57
1/*n*_*F*_(dimensionless)	0.071	0.076	0.091	0.089
*R* ^2^	0.998	0.999	0.999	0.992
*R*_adj_^2^	0.996	0.999	0.999	0.997
*ARE* (%)	1.56	0.54	0.68	1.54
*AIC*	–3.43	–15.72	–16.59	–7.41
Sips				
*q*_*S*_ (mg g^–1^)	19.68	18.64	17.88	16.93
*K*_*s*_ (L mg^–1^)	0.033	0.029	0.027	0.027
*m*_s_ (dimensionless)	0.643	0.654	0.698	0.715
*R* ^2^	0.937	0.996	0.999	0.998
*R*_adj_^2^	0.750	0.980	0.998	0.995
*ARE* (%)	1.85	0.54	0.69	1.55
*AIC*	6.56	–5.72	–6.59	2.58
*q*_*e*_ (mg g^–1^)	19.70	18.54	17.68	16.39

Although Langmuir was not the best model to describe adsorption, the derived *q*_*m*_ parameter is important because it provides a standardized capacity metric across materials reported in the literature. Table 4 summarizes Langmuir maximum adsorption capacities (*q*_*m*_) for Ag^+^ reported in the literature and obtained in this study. The RHSi-1,4-PA adsorbent synthesized in this study achieved a maximum adsorption capacity of 19.94 mg g^–1^ at pH 5.0 and a temperature of 303 K. Although lower than several reported values, this performance is still significant, particularly given the use of a waste-derived adsorbent and mild adsorption conditions, such as a pH close to neutral and room temperature.

**Table 4:** Langmuir Maximum Adsorption Capacities (*q*_*m*_) for Ag^+^ Obtained in This Work and Reported in the Literature, with Experimental Conditions (pH, Temperature, and Initial Concentration *C*_0_) for Comparison

Adsorbent	Source/Preparation method	pH	T (K)	*C*_0_ (mg L^–1^)	Equilibrium time (min)	*q*_*m*_ (mg g^–1^)	Reference
BNS	Bimodal nanoporous silica prepared using polystyrene beads as a physical template and functionalized with aminopropyltriethoxysilane	–	298	5–200	∼50^a^	3.24^b^	^52^
SE-SNPs	Silica nanoparticles modified successively with 3-glycidyloxypropyltrimethoxysilane, branched polyethyleneimine, and sodium 2-bromoethanesulfonate	6	298	20–90	60	21.7	^53^
mag-IIP	Magnetic ion-imprinted polymer synthesized using functionalized magnetite nanoparticles as magnetic core, silver ion as template, N,N-bis(salicylidene)ethylenediamine as ligand, 4-vinylpyridine as functional monomer, and ethylene glycol dimethacrylate as cross-linker	4.0	–	50	10	28.2^c^	^54^
MP-silica	Mesoporous silica synthesized using citric acid as a template and modified with 3-mercaptopropyltrimethoxysilane	–	–	10–200	30	114.9	^55^
Silica	Commercial silica	6.25	298	5–50	∼500^a^	1.83^c^	^56^
MA	Mesoporous alumina synthesized by the hydrothermal method, with P123 as template agent and Al(NO_3_)_3_·9 H_2_O as aluminum source	6–7	298	100–500	–	37.8^c^	^57^
SA-MA	Mesoporous alumina modified with succinic acid	6–7	298	100–500	∼50^a^	85.89	^57^
CNC	Cellulose nanocrystals extracted from cellulose sludge by acid hydrolysis	6.39	298	107.8	–	34.35^c^	^58^
CNF	Cellulose nanofibers prepared by ultrafine grinding from cellulose sludge	5.45	298	107.8	–	15.45^c^	^58^
ChNC	Chitin nanocrystals obtained from crab shell residue by alkali treatment, bleaching, and acid hydrolysis	6.63	298	107.8	–	19.8^c^	^58^
CB	Biochar obtained from commercial cotton subjected to treatment with a 17.7% NaOH solution followed by pyrolysis at 320 °C	3.5	–	10–200	30	19.49	^59^
RHB	Biochar obtained from rice husks subjected to treatment with concentrated HF followed by pyrolysis at 480 °C	3.5	–	10–200	30	10.84	^59^
ARHB	Activated biochar obtained from chemical activation of RHB with ZnCl_2_ at 700 °C	3.5	–	10–200	30	23.11	^59^
SBBs	Silica gel synthesized from sodium silicate and H_2_SO_4_ through sol–gel process and functionalized with 3-mercaptopropyltrimethoxysilane	6	298	96–1436	60	75.8	^60^
RHSi-1,4-PA	Silica obtained from rice husk and functionalized with phthaloyl diamide ligand	5.0	303	20–200	15	19.94	This study

a Value not clearly reported by the authors; instead, it was obtained based on direct evaluation of the kinetic data.

b Value calculated considering the molar mass of Ag.

c *q*_*e*_ value (*q*_*m*_ value not reported).

### 3.5 Thermodynamic Analysis

The thermodynamic parameters of Ag^+^ adsorption onto RHSi-1,4-PA were evaluated using the Van’t Hoff approach to elucidate the spontaneity and heat effects of the process. The thermodynamic equilibrium constants (*K*_*D*_^*°*^) were obtained from the Freundlich isotherm model and converted to dimensionless form to ensure thermodynamic consistency.

The Van’t Hoff plot exhibited good linearity (*R* ^2^ = 0.982), and the obtained thermodynamic parameters are shown in Table 5. The negative *ΔG* ^*°*^ values obtained at all temperatures indicate that the adsorption of Ag^+^ onto RHSi-1,4-PA is spontaneous and thermodynamically feasible. The negative *ΔH* ^*°*^ value confirms the exothermic nature of the adsorption, which is consistent with the decrease in the Ag^+^ uptake observed at higher temperatures. The magnitude of the standard enthalpy variation (*ΔH* ^*°*^ < 40 kJ mol^–1^) indicates that the process is physisorption-dominated.^61^ Furthermore, the negative *ΔS* ^*°*^ value reflects a decrease in the randomness at the solid–liquid interface, which may result from partial dehydration of Ag^+^ and the ordered association of ions at the amide/carbonyl binding sites.^62,63^

**Table 5:** Thermodynamic Parameters of Ag^+^ Adsorption onto RHSi-1,4-PA

*T* (K)	*ΔG* ^*°*^ (kJ mol^–1^)	*ΔH* ^*°*^ (kJ mol^–1^)	*ΔS* ^*°*^ (kJ K^–1^ mol^–1^)
303.15	–9.36 ± 0.02	–6.94 ± 0.33	–0.008 ± 0.001
313.15	–9.44 ± 0.05		
323.15	–9.52 ± 0.02		
333.15	–9.60 ± 0.02		

### 3.6 Proposed Adsorption Mechanisms

The adsorption mechanism governing the interaction between the Ag^+^ ions and the active sites of the RHSi-1,4-PA adsorbent involve both weak coordination and electrostatic and dispersion interactions. In our previous work,^64^ we conducted a comprehensive theoretical and mechanistic investigation of the Ag^+^ adsorption on phthaloyl-modified rice husk-derived silica and proposed an adsorption mechanism involving the formation of weak but specific O→Ag^+^ coordination through the polarized carbonyl groups of the functionalized surface, followed by additional Ag^+^ accumulation to the primary layer via electrostatic and dispersion forces.

This interpretation aligns with the results of FTIR and pH_PZC_ characterization, alongside the pH effect on the adsorption behavior, and equilibrium and thermodynamic analysis. The FTIR spectra (Figure 7) revealed the successful covalent attachment of the phthaloyl diamide ligands to the silica matrix, introducing polarized carbonyl groups that enable interaction with the Ag^+^ ions. The point of zero charge (pH_PZC_ = 6.3) indicated that the RHSi-1,4-PA adsorbent has a net positive surface charge under all the evaluated pH conditions (pH 2–6). Even so, the adsorbent still exhibits considerably high removal (maximum at pH 5, Figure 9), suggesting the contribution of adsorption mechanisms other than electrostatic. The best fit observed for the Freundlich model suggests adsorption on a heterogeneous surface, which is consistent with the coexistence of active sites associated with the polarized carbonyl groups and the weaker, nonspecific adsorption sites that accommodate the subsequent Ag^+^ accumulation. Lastly, the ΔH° value (−6.94 kJ mol^–1^, Table 5) suggests labile, weakly coordinated Ag–O interactions.

Overall, the adsorption of Ag^+^ ions on the RHSi-1,4-PA adsorbent involves labile, weakly coordinated Ag–O interactions with the polarized carbonyl oxygen atoms of the abundant amide groups, followed by weaker secondary accumulation. Figure 12 shows a schematic diagram of the proposed adsorption mechanism.

**Figure 12:**
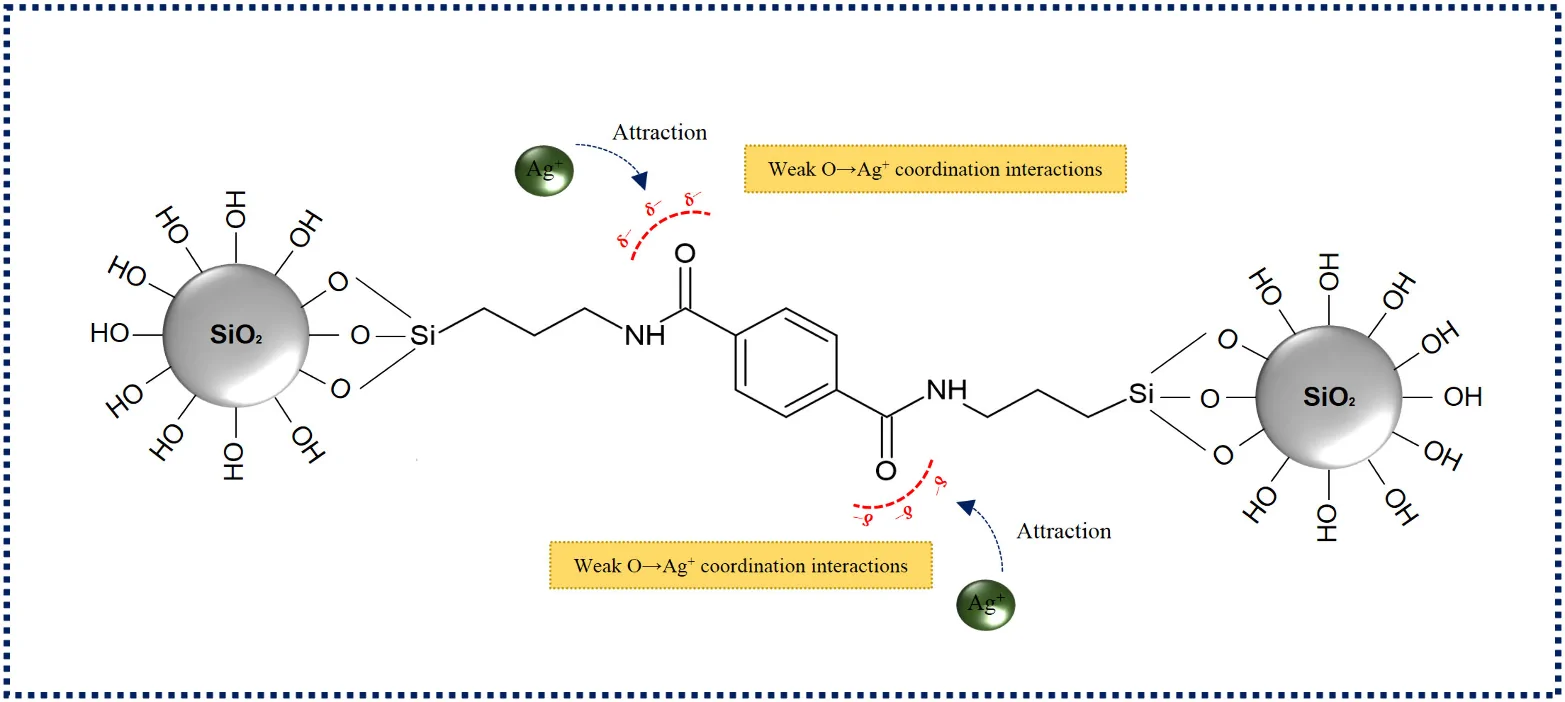
Proposed adsorption mechanism of Ag^+^ on the RHSi-1,4-PA adsorbent.

### 3.7 Adsorbent Regeneration and Reusability

The regeneration of adsorbent materials is key to the economic and environmental sustainability of metal-ion removal processes. Thus, the stability and reusability of RHSi-1,4-PA were evaluated using different mineral acids (HNO_3_, H_2_SO_4_, and HCl) at concentrations of 0.5 and 1.0 mol L^–1^ as regenerating agents. Figure 13 shows the results of equilibrium adsorption capacity (*q*_*e*_) in the second cycle of adsorption.

**Figure 13:**
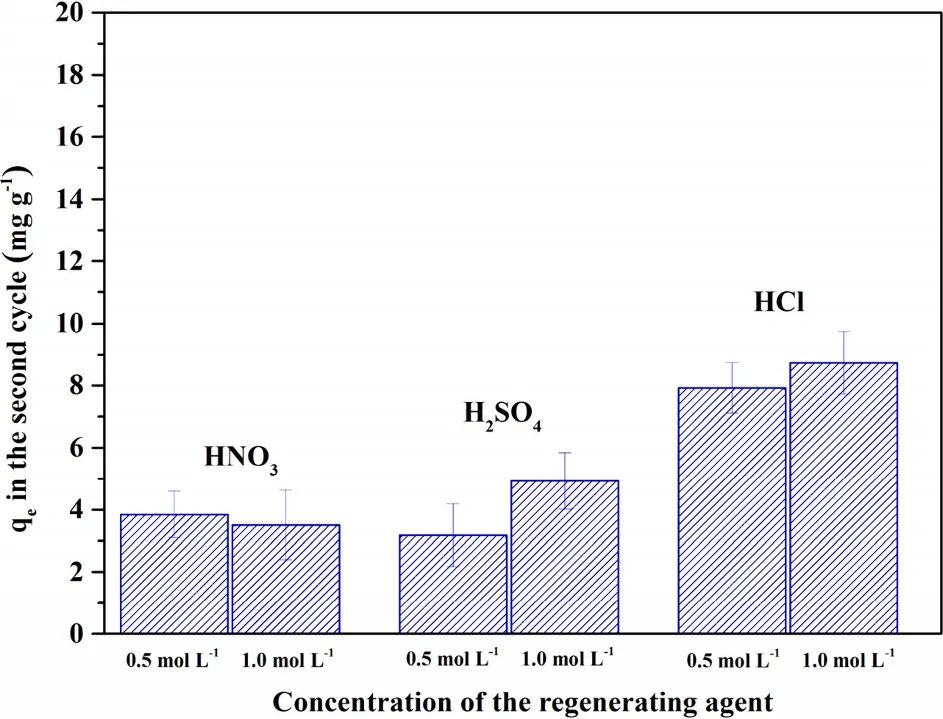
Regeneration and reusability tests: adsorption capacity after regeneration.

It was found that HCl stood out among the three regenerating agents tested. The superior performance of hydrochloric acid may be due to the formation of insoluble AgCl. Nevertheless, the Ag^+^ adsorption capacity in the second adsorption cycle decreased by around 50% for both HCl concentrations evaluated. This behavior suggests that a significant fraction of Ag^+^ ions may remain bound to active sites of the adsorbent surface, or that partial hydrolysis of the grafted ligand may occur under acidic conditions, reducing the number of active sites.

The limited performance of the RHSi-1,4-PA adsorbent in the second Ag^+^ adsorption cycle highlights the need for more efficient regeneration approaches. Reske et al.^5^ found that the Ag^+^ desorption from a zeolite composite was only possible with a 10 mol L^–1^ HCl solution. However, the authors found that this drastic condition also resulted in the removal of the zeolite surface, compromising its reuse in subsequent adsorption cycles. Therefore, using such a high HCl concentration could likely also lead to even more noticeable hydrolysis of the ligand grafted to the silica surface. In addition, regenerating the adsorbent with an acidic solution of such high concentration is not environmentally sound.

### 3.8 Antibacterial Activity

Since it was not possible to regenerate and reuse the RHSi-1,4-PA adsorbent in subsequent adsorption cycles, finding a destination for the solid after adsorption is of utmost importance. Thus, in the present work, the Ag^+^-loaded RHSi-1,4-PA (named RHSi-1,4-PA–Ag^+^) was tested for its antibacterial activity against *Staphylococcus aureus* and *Escherichia coli*. To obtain the RHSi-1,4-PA–Ag^+^ material, the RHSi-1,4-PA adsorbent was loaded with Ag^+^ under the following conditions: initial Ag^+^ concentration of 200 mg L^–1^, adsorbent dosage of 1 g L^–1^, pH 5, and temperature of 25 °C. This resulted in a material with an Ag^+^ content of approximately 19.7 mg g^–1^. Tests using the pristine adsorbent (RHSi-1,4-PA) were also performed for comparison.

Table 6 shows the effect on bacterial growth for both *Staphylococcus aureus* and *Escherichia coli* after treatment with RHSi-1,4-PA, RHSi-1,4-PA–Ag^+^, and free Ag^+^ ions. For *Staphylococcus aureus*, the viable population decreased during the incubation period with RHSi-1,4-PA, RHSi-1,4-PA–Ag^+^, and free Ag^+^ ions, indicating measurable inhibition. However, under the experimental conditions evaluated, the incorporation of Ag^+^ ions into the functionalized silica matrix did not promote a considerable increase in the antibacterial activity compared to RHSi-1,4-PA (without Ag^+^). For *Escherichia coli*, the viable population increased during the incubation period with RHSi-1,4-PA (without Ag^+^), indicating no measurable antibacterial effect under the conditions evaluated. In contrast, the Ag^+^-loaded adsorbent (RHSi-1,4-PA–Ag^+^) exhibited marked antibacterial activity against *Escherichia coli*. After 4 h of incubation, RHSi-1,4-PA–Ag^+^ reduced the viable population from 4.56 ± 0.01 to 3.30 ± 0.00 log_10_ CFU mL^–1^, corresponding to a Δlog of – 1.26, indicating measurable inhibition. Additionally, the exposure of *Escherichia coli* to free Ag^+^ ions resulted in complete inhibition of bacterial growth after 4 h of exposure, probably due to their direct interaction with bacterial structures. Despite that, the silica-supported Ag^+^ material can offer important advancement in terms of controlled release of Ag^+^ ions and improved biocompatibility by reducing dosing frequency and cytotoxicity.^65,66^

**Table 6:** Log_10_ CFU mL^–1^ of *Staphylococcus aureus* and *Escherichia coli* after Contact with the Evaluated Materials for 0 and 4 h

Treatment	0 h (log_10_ CFU mL^–1^)	4 h (log_10_ CFU mL^–1^)	Δlog^a^
	*Staphylococcus aureus*		
Control (bacteria only)	5.51 ± 0.00	5.54 ± 0.02	+0.03 (growth)
RHSi-1,4-PA	5.53 ± 0.01	5.32 ± 0.01	–0.21 (reduction)
RHSi-1,4-PA–Ag^+^	5.58 ± 0.01	5.36 ± 0.01	–0.22 (reduction)
Free Ag^+^	5.46 ± 0.01	5.21 ± 0.13	–0.25 (reduction)
	*Escherichia coli*		
Control (bacteria only)	5.27 ± 0.01	5.36 ± 0.02	+0.09 (growth)
RHSi-1,4-PA	5.02 ± 0.01	5.18 ± 0.02	+0.16 (growth)
RHSi-1,4-PA–Ag^+^	4.56 ± 0.01	3.30 ± 0.00	–1.26 (reduction)
Free Ag^+^	4.30 ± 0.04	ND^b^	–

a Δlog: difference between the mean log_10_ CFU mL^–1^ values at 4 and 0 h (Δlog values were not calculated for treatments containing ND results).

b ND: no colonies were detected under the experimental conditions.

The obtained results confirmed the antibacterial potential of the Ag^+^-loaded adsorbent against *Escherichia coli* and corroborated that the observed antibacterial activity is directly associated with the Ag^+^ ions, which may interact with cellular components, interfere with essential enzymatic processes, compromise membrane integrity, and interact with intracellular components, including nucleic acids.^67–69^ These mechanisms may contribute to the reduced bacterial viability observed in the present study against *Escherichia coli*; however, they were not directly investigated. The incorporation of Ag^+^ ions into the RHSi-1,4-PA adsorbent therefore adds a significant antibacterial property to the material, enhancing its value for dual-function applications.

The stronger antibacterial activity observed for *Escherichia coli* suggests that this Gram-negative bacterial strain was more susceptible to the Ag^+^-loaded adsorbent than the Gram-positive *Staphylococcus aureus* under the experimental conditions used in this study. This behavior may be associated with structural differences in the bacterial cell envelope. Jung et al.^70^ found a higher efficacy of an Ag^+^ ion solution against the Gram-negative *Escherichia coli* than against the Gram-positive *Staphylococcus aureus*, and attributed this behavior to the thickness of the peptidoglycan layer. Similarly, Feng et al.^71^ showed that Ag^+^ ions cause damage to the membrane, proteins, and DNA in both species, but the structural changes observed for *Escherichia coli* were more pronounced and occurred more rapidly, suggesting a defense system of *Staphylococcus aureus* against the inhibitory effects of Ag^+^ ions.

## 4 Conclusions

Silica from rice husk was successfully synthesized and functionalized with three different phthaloyl diamide ligand isomers, yielding adsorbents capable of adsorbing Ag^+^ ions from aqueous media. In-depth characterization confirmed successful functionalization, with the uniformity and extent of functionalization varying with the ligand isomeric structure. The results of the pH effect tests showed that Ag^+^ adsorption was favored at pH 5.0, with the RHSi-1,4-PA adsorbent outperforming the others. The superior performance observed for the RHSi-1,4-PA adsorbent can be ascribed to the more homogeneous coverage and enhanced functionalization efficiency, and to the wider bite angle of the 1,4-phthaloyl ligand, which facilitates the simultaneous interaction of both oxygen and nitrogen atoms with the Ag^+^ ions. The kinetic study showed that adsorption was very fast, reaching equilibrium in about 15 min, and the kinetic curves were well represented by the pseudo-second-order and Elovich models. The Freundlich isotherm model best described the equilibrium data across 303–333 K. The maximum adsorption capacity was 19.94 mg g^–1^. The thermodynamic parameters (*ΔG* ^*°*^ < 0; *ΔH* ^*°*^ = −6.94 kJ mol^–1^; *ΔS* ^*°*^ = −0.008 kJ K^–1^ mol^–1^) indicated a spontaneous and exothermic process, dominated by physisorption with specific but weak coordination interactions. Additionally, the Ag^+^-loaded adsorbent showed pronounced antibacterial activity against *Escherichia coli*.

Overall, the study demonstrates that rice-derived husk silica functionalized with ligands based on phthaloyl diamide is a promising alternative for Ag^+^ adsorption, with ligand isomer-dependent functionalization efficiency and adsorptive performance. The antibacterial activity of the Ag^+^-loaded adsorbent enhances the value of the synthesized material by enabling dual-function applications.

## Supplementary Material



## Notes

Characterization techniques used for rice husk silica and functionalized rice husk silica (Section S.1); models for kinetic, equilibrium, and thermodynamic calculations (Section S.2) (PDF)

## References

[1] Mustapha L. S., Obayomi O. V., Obayomi K. S. (2026). A comprehensive review on potential
heavy metals in the environment: Persistence, bioaccumulation, ecotoxicology,
and agricultural impacts. Ecol. Front..

[2] Oladimeji T. E., Oyedemi M., Emetere M. E., Agboola O., Adeoye J. B., Odunlami O. A. (2024). Review on the Impact
of heavy metals from industrial
wastewater effluent and removal technologies. Heliyon.

[3] Hama
Aziz K. H., Mustafa F. S., Omer K. M., Hama S., Hamarawfa F., Rahmanac K. O. (2023). Heavy metal pollution in the aquatic
environment: Efficient and low-cost removal approaches to eliminate
their toxicity: A review. RSC Adv..

[4] Padhye L. P., Jasemizad T., Bolan S., Tsyusko O. V., Unrine J. M., Biswal B. K., Balasubramanian R., Zhang Y., Zhang T., Zhao J., Li Y., Rinklebe J., Wang H., Siddique K. H. M., Bolan N. (2023). Silver Contamination
and its toxicity
and risk management in terrestrial and aquatic ecosystems. Sci. Total Environ..

[5] Reske G. D., Pereira H. A., Bernicker M. R., Cargnelutti J. F., Dotto G. L., Castilhos F. De. (2024). Graphite oxide impregnated with lta
zeolite as a new adsorbent to uptake Ag (I) from aqueous solution
and its subsequent reuse as an antimicrobial. Mater. Chem. Phys..

[6] Yu H., Zhang H., Zhang C., Wang R., Liu S., Du R., Sun W. (2024). Advances in
treatment technologies for silver-containing
wastewater. Chem. Eng. J..

[7] Kong J., Gu R., Yuan J., Liu W., Wu J., Fei Z., Yue Q. (2018). Adsorption behavior of Ni­(II) onto
activated carbons from hide waste
and high-pressure steaming hide waste. Ecotoxicol.
Environ. Saf..

[8] Kumar R., Laskar M. A., Hewaidy I. F., Barakat M. A. (2019). Modified
adsorbents
for removal of heavy metals from aqueous environment: A review. Earth Syst. Environ..

[9] Taleb M. A., Kumar R., Barakat M. A., Almeelbi T., Seliem M. K., Ahmad A. (2024). Recent advances in heavy metals uptake
by tailored silica-based adsorbents. Sci. Total
Environ..

[10] Shao P., Liang D., Yang L., Shi H., Xiong Z., Ding L., Yin X., Zhang K., Luo X. (2020). Evaluating
the adsorptivity of organo-functionalized silica nanoparticles towards
heavy metals: Quantitative comparison and mechanistic insight. J. Hazard. Mater..

[11] Kainth S., Sharma P., Pandey O. P. (2024). Green sorbents from agricultural
wastes: A review of sustainable adsorption materials. Appl. Surf. Sci. Adv..

[12] Gkika D. A., Tolkou A. K., Katsoyiannis I. A., Kyzas G. Z. (2025). The adsorption-desorption-regeneration
pathway to a circular economy: The role of waste-derived adsorbents
on chromium removal. Sep. Purif. Technol..

[13] Ahmad F. A. (2023). The use
of agro-waste-based adsorbents as sustainable, renewable, and low-cost
alternatives for the removal of ibuprofen and carbamazepine from water. Heliyon.

[14] Nzereogu P. U., Omah A. D., Ezema F. I., Iwuoha E. I., Nwanya A. C. (2023). Silica
extraction from rice husk: Comprehensive review and applications. Hybrid Adv..

[15] Soltani N., Bahrami A., Pech-Canul M. I., González L. A. (2015). Review
on the physicochemical treatments of rice husk for production of advanced
materials. Chem. Eng. J..

[16] Peres E. C., Favarin N., Slaviero J., Almeida A. R. F., Enders M. P., Muller E. I., Dotto G. L. (2018). Bio-nanosilica
obtained from rice
husk using ultrasound and its potential for dye removal. Mater. Lett..

[17] Arias A. N., Jerez A. L. P., Tesio A. Y., Serrano M. R., Bonini N. A., Parentis M. L. (2025). Optimization of surface silanol groups
in mesoporous
SBA-15 and KIT-6 materials: Effects on APTES functionalization and
CO_2_ adsorption. Microporous Mesoporous
Mater..

[18] Barczak M. (2019). Functionalization
of mesoporous silica surface with carboxylic groups by Meldrum’s
Acid and its application for sorption of proteins. J. Porous Mater..

[19] Hu Y., Drouin E., Larivière D., Kleitz F., Fontaine F.-G. (2017). Highly
efficient and selective recovery of rare earth elements using mesoporous
silica functionalized by preorganized chelating ligands. ACS Appl. Mater. Interfaces.

[20] Kalapathy U., Proctor A., Shultz J. (2000). Production
and properties of fexible
sodium silicate films from rice hull ash silica. Bioresour. Technol..

[21] Yalçin N., Sevinç V. (2001). Studies on silica obtained from rice
husk. Ceram. Int..

[22] Thommes M., Kaneko K., Neimark A. V., Olivier J. P., Rodriguez-Reinoso F., Rouquerol J., Sing K. S. W. (2015). Physisorption of Gases, with Special
Reference to the Evaluation of Surface Area and Pore Size Distribution
(IUPAC Technical Report). Pure Appl. Chem..

[23] Verma C., Singh A., Singh P., D K.
Y. R., Alfantazi A. (2024). Regioisomeric
Effect of heteroatoms and functional groups of organic ligands: Impacts
on coordination bonding and corrosion protection performance. Coord. Chem. Rev..

[24] Nassar M. Y., Ahmed I. S., Raya M. A. (2019). A Facile and tunable approach for
synthesis of pure silica nanostructures from rice husk for the removal
of ciprofloxacin drug from polluted aqueous solutions. J. Mol. Liq..

[25] Chun J., Mo Y., Hwang J., Oh K. K., Lee J. H. (2020). Synthesis of ordered
mesoporous silica with various pore structures using high-purity silica
extracted from rice husk. J. Ind. Eng. Chem..

[26] Affandi S., Setyawan H., Winardi S., Purwanto A., Balgis R. (2009). A facile method
for production of high-purity silica xerogels from bagasse ash. Adv. Powder Technol..

[27] Askaruly K., Korobeinyk A. V., Azat S., Yeleuov M., Taurbekov A., Toshtay K., Tauanov Z., Su X. (2023). The electrochemical
behavior of silica and activated carbon materials derived from the
rice husk waste for li-ion cells. Diam. Relat.
Mater..

[28] Aprilia S., Rosnelly C. M., Zuhra, Fitriani F., Akbar E. H., Raqib M., Rahmah K., Amin A., Baity R. A. (2023). Synthesis
of amorphous
silica from rice husk ash using the sol–gel method: Effect
of alkaline and alkaline concentration. Mater.
Today Proc..

[29] Shrestha D., Nayaju T., Kandel M. R., Pradhananga R. R., Park C. H., Kim C. S. (2023). Rice husk-derived mesoporous biogenic
silica nanoparticles for gravity chromatography. Heliyon.

[30] Zhang S., Dong Q., Chen T., Xiong Y. (2016). Combination of light
bio-oil washing and torrefaction pretreatment of rice husk: Its effects
on physicochemical characteristics and fast pyrolysis behavior. Energy Fuels.

[31] Abdul
Salim Z. A. S., Ismail H., Hassan A., Ismail N. H. C., Hashim F. (2023). Characterisation of high purity rice husk silica synthesised
using solvent-thermal treatment with different concentration of acid
leaching. J. Teknol..

[32] Aliyu A. S., Aliyu U. S., Nyakuma A. M. H. B. B., Liman M. S., Gaya U. I., Dunama A. M., Zira J. D., Liman M. M., Dunama A. M., Zira J. D., Liman M. M. (2025). Synthesis and characterisation
of rice husk and palm fruit bunch silica: Compositional, structural,
and thermal analyses. Biomass Convers. Biorefinery.

[33] Ghanei-Motlagh M., Fayazi M., Ali M., Jalalinejad A. (2016). Application
of magnetic nanocomposite modified with a thiourea based ligand for
the preconcentration and trace detection of silver­(I) ions by electrothermal
atomic absorption spectrometry. Chem. Eng. J..

[34] Yang L., Liu X., Song B., Yang S., Zhang X., Zhao C., Wu Y., Zhang L., Huang Y. (2025). Selective adsorption of silver­(I)
by dimercaptosuccinic acid (DMSA) functionalized polyhedral oligomeric
silsesquioxane (POSS)-based hybrid polymer: Behavior and mechanism. React. Funct. Polym..

[35] Li X. X., Wang Z. Y., Na Y. J., Zhang F., Wang W. J., Li Y., Yan Z. Y. (2025). Synthesis of functional organic polymer with specific
binding sites for efficient adsorption of U­(VI) in aqueous solution. J. Environ. Chem. Eng..

[36] Turanov A. N., Karandashev V. R., Artyushin O. I., Sharova E. V., Genkina G. K. (2017). Adsorption
of palladium­(II) from hydrochloric acid solutions using polymeric
resins impregnated with novel N-substituted 2-(diphenylthiophosphoryl)­acetamides. Sep. Purif. Technol..

[37] Li F., Ding T., Li J., Liu Y., Jia J., Guo Q. (2026). Molecular-level mechanisms in amide-based porous polymers for selective
copper adsorption: A DFT study with environmental applications. Environ. Res..

[38] Zhao X., Zhang S., Bai C., Li B., Li Y., Wang L., Wen R., Zhang M., Ma L., Li S. (2016). Nano-diamond particles functionalized with single/double-arm amide–thiourea
ligands for adsorption of metal ions. J. Colloid
Interface Sci..

[39] Diel J. C., Franco D. S. P., Nunes I., dos S., Pereira H. A., Moreira K. S., Burgo T. A. de L., Foletto E. L., Dotto G. L. (2021). Carbon
nanotubes impregnated with metallic nanoparticles and their application
as an adsorbent for the glyphosate removal in an aqueous matrix. J. Environ. Chem. Eng..

[40] Ezzati R., Azizi M., Ezzati S. (2024). A Theoretical
approach for evaluating
the contributions of pseudo-first-order and pseudo-second-order kinetics
models in the Langmuir rate equation. Vacuum.

[41] Lee H., Fiore S., Berruti F. (2024). Adsorption
of methyl orange and methylene
blue on activated biocarbon derived from birchwood pellets. Biomass and Bioenergy.

[42] Puga A., Moreira M. M., Pazos M., Figueiredo S. A., Sanromán M. Á., Delerue-Matos C., Rosales E. (2022). Continuous adsorption studies of pharmaceuticals in
multicomponent mixtures by agroforestry biochar. J. Environ. Chem. Eng..

[43] Ho Y. S., Mckay G. (1999). Pseudo-second
order model for sorption processes. Process
Biochem.

[44] Wu F. C., Tseng R. L., Juang R. S. (2009). Characteristics of Elovich Equation
used for the analysis of adsorption kinetics in dye-chitosan systems. Chem. Eng. J..

[45] Guarín J. R., Moreno-Pirajan J. C., Giraldo L. (2018). Kinetic study of the
bioadsorption
of methylene blue on the surface of the biomass obtained from the
algae *D. antarctica*. J. Chem..

[46] Giles C. H., Smith D., Huitson A. (1974). A general
treatment and classification
of the solute adsorption isotherm. J. Colloid
Interface Sci..

[47] Foo K. Y., Hameed B. H. (2010). Insights into the modeling of adsorption
isotherm systems. Chem. Eng. J..

[48] Duman O., Ayranci E. (2005). Structural and ionization
effects on the adsorption
behaviors of some anilinic compounds from aqueous solution onto high-area
carbon-cloth. J. Hazard. Mater..

[49] Ayranci E., Duman O. (2006). Adsorption of aromatic
organic acids onto high area activated carbon
cloth in relation to wastewater purification. J. Hazard. Mater..

[50] Duman O., Ayranci E. (2010). Attachment of benzo-crown ethers
onto activated carbon
cloth to enhance the removal of chromium, cobalt and nickel ions from
aqueous solutions by adsorption. J. Hazard.
Mater..

[51] Duman O., Ayranci E. (2006). Adsorption characteristics of benzaldehyde, sulphanilic
acid, and p-phenolsulfonate from water, acid, or base solutions onto
activated carbon cloth. Sep. Sci. Technol..

[52] Sim J. H., Umha H. N., A H. H. S., Sung H. K., Oh S. Y., Lee B.-C., Rengaraj S., Kim Y. (2014). Comparison of adsorptive
features between silver ion and silver nanoparticles on nanoporous
materials. J. Ind. Eng. Chem..

[53] Zhang L., Zhang G., Wang S., Peng J., Cui W. (2017). Sulfoethyl
functionalized silica nanoparticle as an adsorbent to selectively
adsorb silver ions from aqueous solutions. J.
Taiwan Inst. Chem. Eng..

[54] Kazemi E., Mohammad A., Shabani H. (2015). Synthesis and characterization
of
a nanomagnetic ion imprinted polymer for selective extraction of silver
ions from aqueous samples. Microchim. Acta.

[55] Pongkitdachoti U., Unob F. (2018). Simultaneous adsorption
of silver nanoparticles and silver ions on
large pore mesoporous silica. J. Environ. Chem.
Eng..

[56] Li H., Wang M., Li Y., Mo F., Zhu L., Li Z., Xu J., Kong Y., Deng N., Chai R. (2021). Adsorption
characteristics of silver atoms and silver ions on silica surface
in silver nanoparticle hydrosol system. Appl.
Surf. Sci..

[57] Zou G., Bao J. (2025). Adsorption of silver
by succinic acid-modified mesoporous alumina
and its sustained-release antimicrobial application. Mater. Today Commun..

[58] Liu P., Sehaqui H., Tingaut P., Wichser A., Oksman K., Mathew A. P. (2014). Cellulose
and chitin nanomaterials for capturing silver
ions (Ag^+^) from water via surface adsorption. Cellulose.

[59] Vassileva P., Uzunov I., Popova T., Voykova D., Avramova I., Mehandjiev D. (2025). Biochars as a solution for silver
removal and antimicrobial
activity in aqueous systems. Appl. Sci..

[60] Quang D. V., Lee J. E., Kim J. K., Kim Y. N., Shao G. N., Kim H. T. (2013). A gentle method
to graft thiol-functional groups onto
silica gel for adsorption of silver ions and immobilization of silver
nanoparticles. Powder Technol..

[61] Bandar S., Anbia M., Salehi S. (2021). Comparison
of MnO_2_ modified
and unmodified magnetic Fe_3_O_4_ nanoparticle adsorbents
and their potential to remove iron and manganese from aqueous media. J. Alloys Compd..

[62] Alnasra O. A., Khalili F. I., Alhnafat F. A. (2024). Enhanced removal of Pb­(II), Zn­(II)
and Cd­(II) ions by insolubilized humic acid: Characterization and
sorption behaviors. Desalin. Water Treat..

[63] Niu Y., Yu W., Yang S., Wan Q. (2024). Understanding the relationship between
pore size, surface charge density, and Cu^2+^ Adsorption
in mesoporous silica. Sci. Rep..

[64] Franco B. M., Fuhr A. C. F. P., Vieira Y., Lütke S. F., Crissien T. J., Silva L. F. O., Dotto G. L. (2026). Theoretical and
mechanistic investigation on Ag^+^ adsorption using phthaloyl
diamide-modified rice husk-derived silica. Surf.
Interfaces.

[65] Yuliatun L., Mariyam M., Inayah N. N., Mizuki C. A., Fitriyah F., Serunting M. A., Wulanda A. A., Herlina I., Yanti D. D., Syahputra T. S. (2026). Enhanced
antibacterial properties of calcium silicate
based materials through silver doping for endodontic applications. Next Mater..

[66] A S. B., S J. L. S., V L. K., Rahulan K. M., S K. (2026). Harnessing
silica-coated silver nanoparticles for combating multidrug-resistant *Pseudomonas aeruginosa*. Appl. Microbiol.
Biotechnol..

[67] Kedziora A., Speruda M., Krzyzewska E., Rybka J., Łukowiak A., Bugla-Płoskonska G. (2018). Similarities
and differences between
silver ions and silver in nanoforms as antibacterial agents. Int. J. Mol. Sci..

[68] Rai M., Yadav A., Gade A. (2009). Silver nanoparticles as a new generation
of antimicrobials. Biotechnol. Adv..

[69] Marambio-Jones C., Hoek E. M. V. (2010). A review of the
antibacterial effects of silver nanomaterials
and potential implications for human health and the environment. J. Nanoparticle Res..

[70] Jung W. K., Koo H. C., Kim K. W., Shin S., Kim S. H., Park Y. H. (2008). Antibacterial activity and mechanism
of action of the
silver ion in *Staphylococcus aureus* and *Escherichia
coli*. Appl. Environ. Microbiol..

[71] Feng Q. L., Wu J., Chen G. Q., Cui F. Z., Kim T. N., Kim J. O. (2000). A mechanistic
study of the antibacterial effect of silver ions on *Escherichia
coli* and *Staphylococcus aureus*. J. Biomed. Mater. Res..

