# Supplementary material for: Valorization of Rice Husk through the Development of New Materials of Silica Functionalized with Phthaloyl Diamide Ligands: Characterization, Isomer Effect, Ag+ Adsorption Investigation, and Antibacterial Activity
Source: ACS Omega. 2026 Oct 28;11(44):68354–70. doi: 10.1021/acsomega.6c04955 (PMC13756677; doi:10.1021/acsomega.6c04955)
Supplement: Supplementary file 1 [file ao6c04955_si_001.pdf]

Valorization of rice husk through the development of new materials of silica functionalized with phthaloyl diamide ligands: Characterization, isomer effect, Ag^+ adsorption investigation, and antibacterial activity

Brenda Mello Franco[1]; Tauana Fernanda Jantsch Voigt[2]; Juliana Felipetto Cargnelutti[3]; Sabrina Frantz Lütke[1,4]; Cristiano Rodrigo Bohn Rhoden[5], Marcos Leandro Silva Oliveira[6], Luis Felipe Oliveira Silva[6*], Guilherme Luiz Dotto[1*]

[1]Research Group on Adsorptive and Catalytic Process Engineering (ENGEPAC), Federal University of Santa Maria, Av. Roraima, 1000-7, 97105–900 Santa Maria, RS, Brazil.

[2]Graduate Program in Veterinary Medicine, Federal University of Santa Maria (UFSM), Santa Maria, RS, Brazil.

[3]Department of Preventive Veterinary Medicine, Federal University of Santa Maria (UFSM), Santa Maria, RS, Brazil.

[4]Chemistry Department, Federal University of Santa Maria–UFSM, Av. Roraima, 1000-7, 97105–900 Santa Maria, RS, Brazil.

[5]Laboratory of Nanostructured Magnetic Materials (LaMMaN), 97010-491, Franciscan University, Santa Maria, RS, Brazil.

[6]Universidad de La Costa, CUC, Calle 58 # 55–66, Barranquilla, Atlántico, Colombia.

*Corresponding authors:

Luis Felipe Oliveira Silva: lfsoacademico@gmail.com

Guilherme Luiz Dotto: guilherme_dotto@yahoo.com.br

Supplementary material

S.1. Characterization of rice husk silica and functionalized rice husk silica

X-ray fluorescence (XRF) analysis was carried out in an Energy Dispersive X-ray Fluorescence Spectrometer (Shimadzu, Model EDX-720, Japan). Scanning electron microscopy (SEM) was performed on a Tescan MIRA 3 (Czech Republic) electron microscope operating at 10 kV, equipped with energy-dispersive X-ray spectroscopy (EDS).

Fourier transform infrared spectroscopy (FT-IR) was performed on a Shimadzu Prestige-21 spectrophotometer (Japan) using the diffuse reflectance technique with KBr. The FTIR spectra were recorded with 45 scans over the range of 400 to 4000 cm^{-1} at a resolution of 4 cm^{-1}. Powder X-ray diffraction (XRD) analysis was carried out using a diffractometer (Rigaku, Miniflex 300, Japan) with Cu–Kα radiation (λ = 1.54051 Å) at 30 kV and 10 mA. The diffractograms were recorded over the range of $10^{o} \leq 2\theta \leq 100^{o}$, using Step mode with a scan speed of 0.5 s and a scan step of 0.03°.

Thermogravimetric (TGA) and derivative thermogravimetric (DTG) curves were obtained in a thermogravimetric analyzer (Netzsch, STA 449 F3 Jupiter®, Germany). The curves were obtained at temperatures from 20 °C to 800 °C, a heating rate of 25 °C min^{-1}, and an N_2 flow rate of 50 mL min^{-1}.

Nitrogen adsorption/desorption isotherms at 77 K were obtained using a volumetric adsorption analyzer (Micrometrics, ASAP 2020, USA). The specific surface area of the materials was obtained from the Brunauer-Emmett-Teller (BET) method. The total pore volume, mesopore volume, pore size distribution, and average pore size were obtained from the Barrett-Joyner-Halenda (BJH) method.

The point of zero charge (pH_{PZC}) was obtained using the 11-point method. First, a NaCl solution at 0.1 mol L^{-1} was prepared, and its pH was adjusted to 11 initial pH values (from 2 to 12) by adding HCl or NaOH solutions (0.1 mol L^{-1}). Then, 20 mL of the pH-adjusted solutions were added to Erlenmeyer flasks containing 0.02 g of the adsorbent, and the mixture was agitated for 24 h at room temperature and 150 rpm using an orbital shaker (Solab, SL 222, Brazil). After that, the solid was separated from the liquid phase by filtration, and the final pH of the solution was measured. The pH_{PZC} was determined from the initial pH versus the final pH plot [1].

S.2. Kinetic, equilibrium, and thermodynamic calculations

For the kinetic evaluation, models based on the kinetics of chemical reactions were used, including the pseudo-first-order model (PFOM), the pseudo-second-order model (PSOM), and the Elovich model [2], whose mathematical expressions are presented by Equations (1), (2), and (3), respectively:

$$q_t = q_1\left(1 - exp\left(-k_1 t\right)\right) \quad (1)$$

$$q_t = \frac{t}{\left(\frac{1}{k_2 q_2^2}\right) + \left(\frac{t}{q_2}\right)} \quad (2)$$

$$q_t = \frac{1}{\alpha}\ln\left(1 + \alpha\beta t\right) \quad (3)$$

Where q_1 and q_2 (mg g^{-1}) are the adsorption capacities predicted by the PFOM and PSOM, respectively, k_1 (min^{-1}) and k_2 (g mg^{-1} min^{-1}) are the rate constants predicted by the PFOM and PSOM, respectively, β (mg g^{-1} min^{-1}) is the initial velocity of the Elovich

model due to $(dq/dt) = \alpha$, when $qt = 0$, and α (g mg^{-1}) is the desorption constant of the Elovich model.

The adsorption equilibrium was interpreted through isotherms, using the Langmuir [3], Freundlich [4], and Sips [5] isotherm models, given by Equations (4), (5), and (6), respectively:

$$q_e = \frac{q_m K_L C_e}{1 + K_L C_e} \tag{4}$$

$$q_e = K_F C_e^{\frac{1}{nF}} \tag{5}$$

$$q_e = \frac{q_s (K_s C_e)^{m_s}}{1 + (K_s C_e)^{m_s}}$$

(6)

where q_m and q_s (mg g^{-1}) are the maximum adsorption capacities predicted by the Langmuir and Sips models, respectively, K_L (L mg^{-1}), K_F ((mg g^{-1}) (mg $L^{-1})^{-1/nF}$), and K_S (L mg^{-1}) are the equilibrium constants of the Langmuir, Freundlich, and Sips models, respectively, $1/n_F$ (dimensionless) is the heterogeneity factor of the Freundlich model, and m_s (dimensionless) is the exponent of the Sips model.

The thermodynamic parameters standard Gibbs free energy variation ($\Delta G°$), standard enthalpy variation ($\Delta H°$), and standard entropy variation ($\Delta S°$) were determined using Equations (7), (8), and (9) [6,7]:

$$\Delta G^{\circ} = -8.314 T \ln\left(K_D^{\circ}\right) = \Delta H^{\circ} - T \Delta S^{\circ}$$

(7)

$$\ln\left(K_D^\circ\right) = \frac{\Delta S^\circ}{R} - \frac{\Delta H^\circ}{RT}$$

(8)

$$K_D^\circ = \frac{\left(K_F \rho\right)}{1000}\left(\frac{10^6}{\rho}\right)^{\left(1-\frac{1}{n_F}\right)} \quad (9)$$

Where T (K) is the absolute temperature, K_D° is the thermodynamic equilibrium constant, K_F ((mg g^{-1}) (mg L^{-1})$^{-1/nF}$) is the equilibrium constant of the Freundlich isotherm model, $1/n_F$ (dimensionless) is the heterogeneity factor of the Freundlich model, and ρ (g mL^{-1}) is the apparent density.

The parameters of the adsorption kinetics and isotherm models were estimated by nonlinear regression using the Quasi-Newton method. The calculations were performed using Origin software (OriginPro 2016 SR0). The quality of the fits was verified using the coefficient of determination (R^2), the adjusted coefficient of determination (R^2_{adj}), the average relative error (*ARE*), and the Akaike information criterion (*AIC*). The thermodynamic parameters were determined by linear regression, with the R^2 value used to evaluate the fit.

References

(1) Netto, M. S.; Oliveira, J. S.; Salau, N. P. G.; Dotto, G. L. Analysis of adsorption isotherms of Ag^+, Co^{+2}, and Cu^{+2} onto zeolites using computational intelligence models. *J. Environ. Chem. Eng.* **2021**, *9*, 104960. https://doi.org/10.1016/j.jece.2020.104960.

(2) Qiu, H.; Lv, L.; Pan, B. C.; Zhang, Q. J.; Zhang, W. M.; Zhang, Q. X. Critical review in adsorption kinetic models. *J. Zhejiang Univ. Sci. A.* **2009**, *10*, 716–724.

https://doi.org/10.1631/jzus.A0820524.

(3) Langmuir, I. The adsorption of gases on plane surfaces of glass, mica and platinum. *J. Am. Chem. Soc.* **1918**, *40*, 1361–1403. https://doi.org/10.1021/ja02242a004.

(4) Freundlich, H. Über die Adsorption in Lösungen. *Zeitschrift Für Phys. Chemie.* **1907**, *57U*, 385–470. https://doi.org/10.1515/zpch-1907-5723.

(5) Sips, R. On the structure of a catalyst surface. *J. Chem. Phys.* **1948**, *16*, 490–495. https://doi.org/doi.org/10.1063/1.1746922.

(6) Bonilla-Petriciolet, A.; Mendoza-Castillo, D. I.; Reynel-Ávila, H. E. *Adsorption Processes for Water Treatment and Purification*, Springer International Publishing, 2017. https://doi.org/10.1007/978-3- 319-58136-1.

(7) Lima, E. C.; Hosseini-Bandegharaei, A.; Moreno-Piraján, J. C.; Anastopoulos, I. A critical review of the estimation of the thermodynamic parameters on adsorption equilibria. Wrong use of equilibrium constant in the Van' t Hoof equation for calculation of thermodynamic parameters of adsorption. *J. Mol. Liq.* **2019**, *273*, 425–434. https://doi.org/10.1016/j.molliq.2018.10.048.
